# Effects of Co-Exposure to Benzene, Toluene, and Xylene, Polymorphisms of microRNA Genes, and Their Interactions on Genetic Damage in Chinese Petrochemical Workers

**DOI:** 10.3390/toxics12110821

**Published:** 2024-11-16

**Authors:** Shuangqi Li, Xiaojing Liao, Rui Ma, Na Deng, Haimei Wu, Zhaorui Zhang, Liping Chen, Qing Wang, Qilong Liao, Qianxi Li, Xinyi Ouyang, Yongmei Xiao, Qifei Deng

**Affiliations:** 1 Department of Occupational and Environmental Health, School of Public Health, Sun Yat-Sen University, 74 Zhongshan 2nd Road, Yuexiu District, Guangzhou 510080, China; shuangqi0717@163.com (S.L.);; 2 School of Public Health, Guangzhou Medical University, Xinzao Road, Panyu District, Guangzhou 511436, China; 3 Huadu Center for Disease Control and Prevention, Guangzhou 510801, China

**Keywords:** BTX co-exposure, microRNA, genetic polymorphisms, genetic damage, gene–environment interaction, petrochemical workers

## Abstract

Benzene, toluene, and xylene (BTX) co-exist in human environments, yet their individual and combined effects on genetic damage at low exposure levels are not fully understood. Additionally, single nucleotide polymorphisms in microRNAs (mirSNPs) might be involved in cancer etiology by affecting the related early health damage. To investigate the influence of BTX exposure, mirSNPs, and their interactions on genetic damage, we conducted a cross-sectional study in 1083 Chinese petrochemical workers, quantifying the BTX cumulative exposure levels and multiple genetic damage biomarkers. Additionally, we genotyped multiple common mirSNPs. Benzene and a BTX mixture were positive associated with the olive tail moment (OTM) and tail DNA% (*p* < 0.05). Higher levels of toluene and xylene enhanced the association of benzene with genetic damage levels. Genotypes and/or mutant allele counts of miR-4482-related rs11191980, miR-4433-related rs136547, miR-27a-related rs2594716, miR-3130-related rs725980, and miR-3928-related rs878718 might significantly influence genetic damage levels. Stronger effect estimates of benzene/BTX exposure were found in carriers of miR-196a-2-related rs11614913 heterozygotes and of wild homozygotes of miR-1269b-related rs12451747, miR-612-related rs12803915, and miR-4804-related rs266437. Our findings provide further support of the involvement of BTX co-exposure, mirSNPs, and their gene–environment interactions in determining the severity of DNA strand break in a complex manner.

## 1. Introduction

Benzene, toluene, and xylene (BTX), characterized by a single benzene ring, are vital petrochemical materials which are commonly used in the production of a large number of petrochemicals, ranging from various solvents to drugs. Due to their growing significance in producing everyday products, BTX have a vast and steadily growing market. According to “BTX Market Size & Share Analysis-Growth Trends & Forecasts (2024–2029)” [1], the global annual demand for BTX is nearly 108 million metric tons, with a demand growth rate of approximately 5%, and the global BTX market is estimated to register a compound annual growth rate (CAGR) of more than 3.5% by 2028. Owing to the dramatic increase in the production and usage of BTX and their related downstream products, volatile BTX that are consequently released are ubiquitously distributed in various human living and working environments. As a major player in the Asia-Pacific region’s BTX market, China’s growing petrochemical industry has led to rising BTX pollution levels, posing significant health risks to populations exposed to these chemicals both at work and in the environment [2].

After entering into the body, mainly through inhalation, BTX compounds can be metabolized by cytochrome P450 (CYP) and other enzymes to generate reactive metabolites to cause multiple adverse health effects, ranging from neurological impairment to cancers [3,4,5]. Thus, BTX are one of the focuses of great concern in view of their deleterious health effects associated with chronic or acute exposure [3,4,5]. Benzene has received more attention than the other BTX components, as there is conclusive evidence to support the assertion that it has strong genotoxicity and can cause cancers such as leukemia and lymphohematopoietic malignancies in chronically exposed humans, even at relatively low levels of exposure [5]. Thus, benzene has been classified as a known human carcinogen (Group 1) by the International Agency for Research on Cancer (IARC) [6,7]. Toluene and xylene, on the other hand, have been the subject of relatively limited and not fully conclusive research [3,4]. Even so, their effects on genetic damage, the most critical early biologic event for cancer development, have attracted more and more attention in recent years. Furthermore, since toluene and xylene frequently co-exist with benzene in various human environments and share similar enzyme-mediated biotransformation, their simultaneous exposure might alter benzene metabolism and then affect its toxic effects [8]. Previous research in humans and mice suggests that simultaneous exposure to high levels of benzene and toluene (≥50 ppm) can impact benzene metabolism and increase benzene-related genetic damage [9]. However, different co-exposure dose ranges demonstrate disparate effects, which might be caused by the dose-dependency of benzene metabolism [9]. With more effective control actions having been implemented to reduce airborne BTX (especially benzene) levels to close to or below the occupational and community air standards, their health effects and underlying mechanisms at lower co-exposure levels have attracted significant attention [10,11,12]. We previously evaluated the effects of BTX co-exposure on pulmonary function [13] and declines in hematologic parameters [10] in Chinese petrochemical workers with long-term low-dose exposure. Research on genetic damages utilizing low co-exposure levels would further characterize the benzene/toluene/xylene dose–response relations and their interactions and provide more important data for risk assessment and management in relation to the public [12].

In addition to environmental hazards, genetic damage levels are also determined by individual genetic predisposition [14]. Studying genetic determinants of environmental health effects can provide a scientific basis for identifying high-risk groups within exposed populations. However, data on genetic factors conferring susceptibility or resistance to BTX-related genotoxicity is relatively limited, with most research focused on polymorphisms in metabolic enzyme genes and DNA damage repair genes [15]. Thus, exploring additional genetic factors linked to BTX-related genotoxicity is essential. MicroRNAs (miRNAs) are highly conserved endogenous noncoding RNAs that post-transcriptionally regulate gene expressions through translational inhibition or mRNA degradation [16]. MiRNA-mediated gene expression regulations are important for the cellular response to environmental stress and genetic damage, and therefore are extensively implicated in various human diseases like cancers [17]. The miRNA biogenesis process begins with pri-miRNA transcripts, which are cut to form pre-miRNAs and then mature into miRNA duplexes after cytoplasmic export. The mature miRNA duplex’s 5p or 3p strands pair with argonaute proteins to create an RNA-induced silencing complex that targets mRNA for expression suppression, with targeting specificity determined by the miRNA seed sequence’s complementarity to the mRNA’s ‘seed-match’ region [16]. It has been demonstrated that any sequence alteration in miRNA genes (including pri-miRNAs, pre-miRNAs, mature miRNAs, and seed regions), especially single nucleotide polymorphisms (SNPs, i.e., mirSNPs), can alter the biogenesis, activity, or bioavailability of the corresponding miRNAs, and eventually exert significant impacts on the expression and/or functions of numerous targets [18]. Thus, mirSNPs are important genetic determinants for individual variations in the cellular response to environmental hazards, phenotypes, and disease susceptibility [19]. A large number of epidemiological studies have demonstrated that mirSNPs are associated with cancer risk [20]. Our previous study in Chinese coke oven workers elucidated that rs11614913, located in the mature sequence of miR-196a2, and its interactions with environmental factors might affect oxidative damage levels, a type of cancer-related early health damage [21]. This preliminarily suggested that mirSNPs might be involved in cancer etiology by affecting the related early health damage. However, most genetic variations known to be located within miRNA genes according to the databases for miRNA-related SNPs (i.e., miRNASNP) have yet to be tested for their links to cancer-related early health damage, especially genetic damage, in the contexts of BTX exposure.

Given that understanding the effects of low-dose BTX co-exposure, mirSNPs, and their interactions on genetic damage levels is important for the development of early prevention strategies for exposed populations, we conducted a preliminary cross-sectional study in Chinese petrochemical workers with long-term occupational exposure primarily to low-dose BTX components. We previously conducted a follow-up study in these workers to evaluate the hematological effects of low-dose BTX co-exposure [10]. In the present study, we quantified their occupational BTX exposure levels by calculating cumulative exposure (CE), measured many genetic damage indices, and genotyped multiple common mirSNPs which were screened out from the miRNASNP database in the baseline stage. We separately evaluated the individual effects of BTX components and SNPs on genetic damage levels, as well as the BTX interactions and the gene–environment interactions. The present study may further enhance our knowledge about the environmental and genetic determinants of the severity of genetic damage and provide further theoretical underpinning for the health surveillance and early screening of BTX-exposed populations.

## 2. Materials and Methods

### 2.1. Study Subjects

As described previously [10], we recruited a total of 1443 BTX-exposed workers from two large state-owned petrochemical plants located in Guangzhou and Maoming, respectively, in southern China. Participants were selected based on the following criteria: (1) workers who had been employed in workplaces where BTX were the primary occupational hazards for at least one year; (2) workers without a history of serious diseases, such as tumors, cardiopulmonary diseases, or chronic immune diseases; (3) workers who had not taken medicine or undergone X-ray examinations in the week before the survey; and (4) workers who had completed the occupational questionnaire and physical examinations and had provided some biological sample materials.

In the present study, we further excluded non-Han subjects and those without sufficient heparin-anticoagulated peripheral venous blood samples for the measurement of genetic damage biomarkers. Finally, a total of 1083 workers were selected in this study. All participants understood the purpose and significance of this study and signed written informed consent forms. They were then interviewed by trained personnel using a pre-tested occupational questionnaire and provided information including their demographic characteristics, lifestyle habits (such as smoking and alcohol consumption), personal and family history of serious diseases, and occupational experience (such as workplaces, type of work, and duration of exposure). Individuals who smoked ≥1 cigarette/day for ≥1 year were defined as smokers, and those who consumed alcohol at least once a week for ≥1 year were defined as alcohol drinkers. After the interview, we collected from each subject ~5 mL of heparin sodium-anticoagulated fasting elbow venous blood for single-cell gel electrophoresis (SCGE) and the cytokinesis-block micronucleus (CBMN) assay. We also collected ~2 mL of ethylenediaminetetraacetic acid (EDTA)-anticoagulated venous blood from each subject. However, after the detection of hematologic parameters [10] and other indicators (such as blood biochemical parameters), only 667 subjects had sufficient EDTA-anticoagulated blood samples for DNA extraction. Thus, there were only 667 subjects in the mirSNP analysis.

### 2.2. Individual BTX Exposure Assessment

To assess long-term occupational BTX exposure, we calculated BTX CE levels for all participants in the present study as mentioned in our previous study [10]. In brief, we monitored ambient BTX concentrations in the workplaces over the three years preceding the study. BTX concentrations were measured using thermal desorption and capillary gas chromatography coupled with hydrogen flame ionization detector following the protocols and quality control methods recommended by the National Institute for Occupational Safety and Health (NIOSH) method 1501. The concentrations below the limits of detection (LOD) were replaced by LOD/2. We calculated 8 h-TWA concentrations for BTX components and then calculated CE levels (mg/m^3^ × year) by multiplying the three-year mean 8 h-TWA concentrations in workplaces by work years.

### 2.3. CBMN Cytometry Assay

We performed the CBMN cytometry assay to evaluate chromosome damage levels using the standard method described by Fenech [22] with minor modifications. We added 0.5 mL of heparin sodium-anticoagulated blood to the prepared 1640 culture medium which contained bovine serum and phytohemagglutinin, and incubated the cultures at 37 °C with a carbon dioxide concentration of 5% for 44 h. Then, we added cytochalasin B (6 μg/L) into the cell culture medium and continued to incubate for another 28 h. Cell cultures were centrifuged at 1500 rpm for 10 min in a low-temperature centrifuge at 4 °C to remove the supernatant culture medium. We treated cells with 5 mL of 0.075 mol/L KCl to lyse red blood cells, and then removed the supernatant. We repeatedly added 5 mL of fixative consisting of methanol/acetic acid (3:1) into the culture tube, centrifuged the mixture at 1200 rpm for 10 min, and then discarded the supernatant. After the last centrifugation, we retained about 150 μL of liquid and resuspended the cell pellet. The suspension was dropped onto a clean glass slide, stained with a 10% Giemsa staining solution, and finally observed under a microscope. The frequency of micronuclei (MN), nucleoplasmic bridges (NPBs), and nuclear buds (NBUDs) were evaluated according to Fenech’s criteria [23].

### 2.4. SCGE Assay

We performed the SCGE assay to evaluate DNA strand breakage levels using the standard method described by Singh [24] with some modifications. We centrifugated 3 mL of heparin sodium-anticoagulated blood to separate white blood cells, and then used a lymphocyte separation solution to separate lymphocytes, which were washed with phosphate-buffered saline (PBS) to adjust the cell concentrations to 106–107 cells/mL. The slides were immersed in 75% alcohol for a whole night. After being air-dried, the frosted glass slides were washed twice with double distilled water (ddH_2_O). When the glass slides were dry, the first layer was covered with melted typical-melting-point agarose (NMPA) and kept at 4 °C to be solidified. Then, 100 µL of the lymphocyte suspension was mixed thoroughly with 100 µL of 0.5% low-melting-point agarose (LMPA) at 37 °C, which was then used to quickly cover the first agarose layer. The slides were then immersed in a lysing solution (2.5 M NaCl, 100 mM Na2-EDTA, 10 mM Tris-base, 5 g sodium sarcosinate, pH 10, and 1% TritonX-100, 10% DMSO added fresh) at 4 °C for overnight to lyse the cells and permit DNA unfolding. Afterward, the slides were washed twice with PBS and put in a 0.3 mol/L NaOH buffer at 4 °C for 20 min to allow DNA unwinding. Electrophoresis was conducted for the next 20 min at 300 mA and 25 V using a horizontal electrophoresis tank at 4 °C. After electrophoresis, the slides were immersed in the Tris-HCL neutralization buffer for 10 min and washed gently. Then, the slides were stained by 50 μL of 2 μg/mL PI staining solution for 25 min in the dark. After staining, slides were rinsed in distilled water and observed (within 24 h) under a fluorescence microscope equipped with an emission wavelength of 590 nm and an excitation wavelength of 549 nm. We used the comet assay software project 1.2.3beta1(CASP), (https://casp-uk.net/, accessed on 1 December 2013) to analyze randomly photographed pictures and calculated the olive tail moment (OTM), the percentage of tail DNA (Tail DNA%), and tail moment of the comet after observing 50 cells in each slide. All of these steps were performed in the dark to avoid additional DNA damage [24].

### 2.5. Selection and Genotyping of mirSNPs

Based on the miRNASNP v2.0 (https://ngdc.cncb.ac.cn/databasecommons/database/id/1681, accessed on 1 December 2013) a solid public database providing miRNA-related SNPs, we performed an integrative bioinformatic analysis to screen out SNPs on the seed sequences, mature sequences, and precursor sequences for all human miRNAs in the miRBase19.0 database (http://www.mirbase.org/, accessed on 1 December 2013). We selected the most promisingly functional SNPs based on a series of criteria: (1) common SNPs with a minor allele frequency (MAF) ≥ 0.05 in the Chinese Han population (CHB) were chosen; (2) SNPs located on the miRNA seed sequences or mature miRNA sequences were prioritized; and (3) for SNPs located on miRNA precursor sequences, we performed an extensive literature review and only selected the SNPs who or whose corresponding miRNAs were reported to be associated with leukemia pathogenesis, toxic metabolism, genetic damage, and/or related mechanisms. Finally, a total of 61 mirSNPs were selected.

We used the iPLEX system (Sequenom) (https://www.cd-genomics.com/sequenom-massarray-iplex-gold.html, accessed on 1 December 2013) for SNP genotyping. Before the Sequenom experiments, we used Sequenom’s primer design software Assay design 3.1 (Laboratory Corporation of America Holdings, Burlington, NC, USA) to design the PCR reaction and single-base extension primers for the selected mirSNPs according to their sequence information. The primers for 46 mirSNPs were successfully designed (success rate was 75.41%) (Table S1), while the primer design for the other 15 mirSNPs failed (Table S2). Then, we replaced the 15 mirSNPs (called original mirSNPs) with other SNPs (Table S2) according to the following criteria: (a) the SNPs were located in the regions from 1000 kb upstream to 1000 kb downstream of the original mirSNPs; (b) the SNPs were in high linkage disequilibrium (LD) (i.e., r2 ≥ 0.8) with the original mirSNPs; (c) the primers for SNPs can be successfully designed; and (d) when multiple SNPs met the above three criteria, the strongest LD SNP was prioritized.

A total of 667 subjects with sufficient EDTA-anticoagulated blood samples were included in the mirSNP analysis. Genomic DNA was extracted from 400 μL of EDTA-anticoagulated blood samples using DNA extraction kit (Tiangen, Beijing, China) according to the manufacturer’s protocol. The selected 61 SNPs were genotyped using Sequenom MassArray system (Beijing Bomiao Biological Co., Ltd., Beijing, China). For each selected SNP, 10% of samples were further randomly selected for repeated genotyping, and the results were 100% concordant.

### 2.6. Statistical Analyses

BTX CE levels, OTM, tail DNA%, and tail moments were natural logarithm (ln)-transformed to obtain a normal distribution. All these ln-transformed biomarkers were standardized before statistical analyses to improve the comparability of the effect estimates. Unless otherwise stated, we adjusted for several primary variables in the statistical analyses, including age (continuous), gender (male/female), smoking status (smokers/non-smokers), pack-years of smoking (continuous), drinking status (drinkers/non-drinkers), factory location (Guangzhou/Maoming), and body mass index (BMI, calculated as weight in kilograms divided by height in meters squared) (continuous).

We divided the participants according to their general characteristics, including age (≤40 vs. >40, which was the median age), gender (female vs. male), smoking status (non-smokers vs. smokers), drinking status (non-drinkers vs. drinkers), and BMI (<24 kg/m^2^ vs. ≥24 kg/m^2^). We evaluated the between-characteristic differences in genetic damage levels via multivariable covariance analysis. In addition, we used covariate-adjusted generalized linear models to evaluate the associations of individual BTX (as independent variables) with genetic damage indicators (as dependent variables) in the total population. For benzene, which was observed to be associated with genetic damage biomarkers, we further assessed the between-characteristic differences in its association by adding an interaction term of benzene CE levels (continuous) and general characteristics (categorical) into covariate-adjusted generalized linear models.

Then, we used generalized weighted quantile sum (gWQS) models and interaction analysis to assess the impact of BTX co-exposure on genetic damage indices. The weighted quartile sum (wqs) index for the three BTX components was divided into four quartiles, and the dataset was randomly split into training (40%) and validation sets (60%), with significance testing for β based on independent data, and we used 10,000 bootstrap iterations to enhance prediction sensitivity and stability. The contribution of each BTX component was evaluated by its weight in the models. For components that were not found to be associated with genetic damage, we further evaluate their modifying effects on benzene’s genetic damage effects. We divided the subjects into three exposure groups according to the tertile CE levels of toluene and xylene, respectively. Then, we assessed their modifying effects on the associations of benzene with genetic damage by adding an interaction term of benzene CE levels (continuous) and exposure groups (continuous and categorical) into covariate-adjusted generalized linear models.

All SNPs were tested for the Hardy–Weinberg equilibrium (HWE) in the total population by a goodness-of-fit Chi-square test. The between-genotype differences in genetic damage levels were examined by means of multivariate analysis of covariance. We assessed the associations of the number of variant alleles of SNPs (as the independent variable) and genetic damage levels (as the dependent variable) in the total population using covariate-adjusted multivariate linear regression models. To explore the gene–environment interactions of benzene and the BTX mixture with SNPs, we evaluated their mutual effect modification on each other’s associations with genetic damage by adding their interaction terms (including those of environment factors (continuous) and genotypes (categorical) and those of the number of SNP variant alleles (continuous) and exposure groups (categorical)) into covariate-adjusted generalized linear models.

All data analyses were carried out in RStudio software (version 4.1.2) and SPSS 26.0 (SPSS, Chicago, IL, USA). Two-sided *p* < 0.05 was considered statistically significant.

## 3. Results

### 3.1. Subject Characteristics

Table 1 shows the general characteristics, BTX CE levels, and genetic damage indices in the 1083 participants in the present study. Subjects had a mean age of 40.01 years, the majority of them were male (73.68%), and most workers were non-smokers (68.79%). About half of the subjects consumed alcohol (45.43%). More than half of the subjects were working in Maoming (65. 28%) and had a relatively long occupational BTX exposure history (mean work years: 19.21). Their average BMI was 23.20 kg/m^2^. Their median CE levels of benzene, toluene, and xylene were 0.66, 0.78, and 1.46 mg/m^3^ × year, respectively. The median frequencies of MN, NPB, and NBUD were 9, 1, and 2 per 1000 cells, respectively. The median levels of OTM, tail DNA%, and tail moments were 0.35, 1.50, and 0.13, respectively. We then compared the genetic damage levels between subjects with different general characteristics, and found that females had higher MN frequency, OTM, and tail moments after adjusting for other primary covariates (*p* < 0.05) (Table S3).

Since 416 subjects in the total population were not tested for SNPs due to their limited EDTA-anticoagulated blood samples, we also compared their general characteristics, BTX CE levels, and genetic damage indices with those who were included in subsequent mirSNP analysis (Table S4). We found that there were significant differences in terms of most of the general characteristics between these two populations (*p* < 0.05). However, BTX CE levels and genetic damage levels were not significantly different after adjusting for these primary covariables (all *p* > 0.05) (Table S4).

### 3.2. Individual and Combined Effects of BTX Components with Genetic Damage

We first divided BTX into two groups based on the mean values and compared the levels of genetic damage between the high-exposure and low-exposure groups (Table 2). In the high-benzene and high-xylene groups, tail DNA% was significantly higher than in the low exposure groups (*p* < 0.05). We further estimated the associations of single BTX components with genetic damage indices. We found that a 1-SD increase in ln-transformed benzene CE levels was associated with a 0.048-SD increase in ln-transformed OTM (95% CI: 0.007, 0.090) and a 0.073-SD increase in ln-transformed tail DNA% (95% CI: 0.027, 0.119) in the total population (Table 2). These associations of ln-transformed benzene with genetic damage indices were more pronounced in subjects older than 40 years (all *P*_interaction_ < 0.05) (Table S5). Consistently, benzene was also significantly positively correlated with OTM and tail DNA% (*p* < 0.05) in subjects included in the mirSNP analysis (Figure S1). However, we did not find any significant associations of BTX components with tail moments and the indicators of chromosomal damage (all *p* > 0.05) (Table 2), thus we did not consider these indicators in the subsequent analyses.

**Figure 1:**
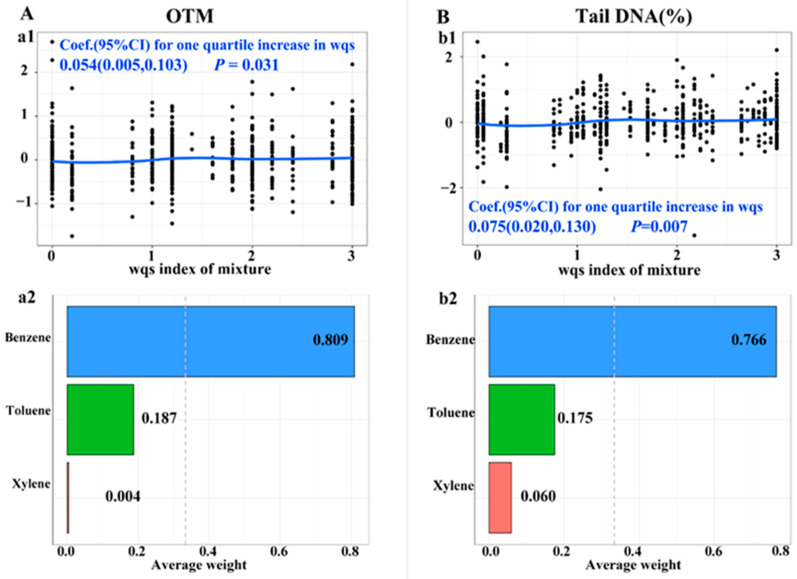
The dose–effect relationship of co-exposure to the BTX mixture (**a1**,**b1**) with (**A**) OTM and (**B**) tail DNA%, as well as the contribution of individual BTX components (**a2**,**b2**). Abbreviations: wqs, weighted quartile sum index for BTX mixture; Coef. (95% CIs), coefficients and 95% confidence intervals; OTM, olive tail moment; Tail DNA%, percentage of DNA in the comet tail. The gWQS models were adjusted by age, gender, smoking status, pack-years of smoking, drinking status, factory location, and BMI.

**Figure 2:**
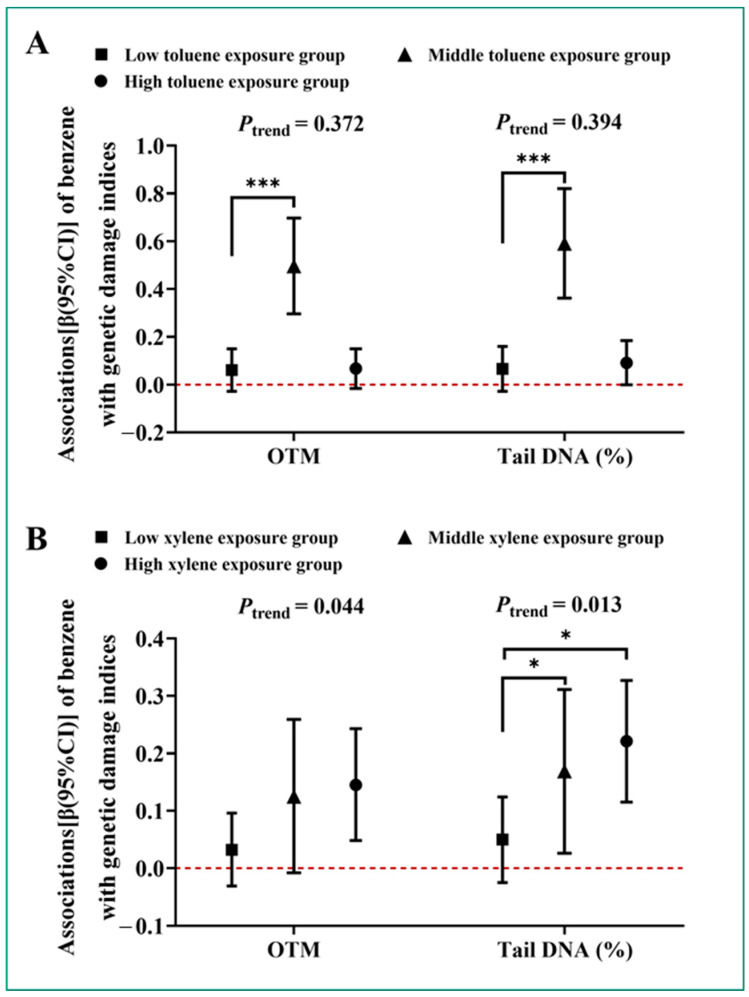
Modifying effects of (**A**) toluene and (**B**) xylene on the associations [β (95% CI)] of benzene with OTM and tail DNA%. Abbreviations: OTM, olive tail moment; Tail DNA%, percentage of DNA in the comet tail. The lines represent the associations of benzene CE levels with genetic damage indices which were analyzed by generalized linear models adjusted for age, gender, smoking status, pack-years of smoking, drinking status, factory location, and BMI. The red dashed line represents the baseline (no association, β = 0). Significant levels are annotated as *** *P*_interaction_ < 0.001 in comparison to the low-exposure group and * *P*_interaction_ < 0.05 in comparison to the low-exposure group.

The combined effects of BTX components with OTM and tail DNA% in gWQS models are shown in Figure 1. A quartile increase in the wqs index of ln-transformed BTX was associated with a 0.054 increase in OTM (95% CI: 0.005, 0.103) and a 0.075 increase in tail DNA% (95% CI: 0.020, 0.130), respectively. The main contributor with the highest weight was benzene, with its weighted percentage being 0.809 in OTM and 0.766 in tail DNA%. Although the individual effects of toluene and xylene on genetic damage levels and their contributions to BTX’s combined effects were limited, we further investigated their modifying effects on the associations of benzene. As shown in Figure 2, we observed that in comparison to the low-exposure groups, the associations of benzene CE levels with OTM and tail DNA% were significantly stronger in subjects with medium toluene exposure levels and in workers with medium and higher xylene exposure levels (*P*_interaction_ < 0.05). Interestingly, the magnitude of these associations was significantly increased with the increase in xylene exposure levels (*P*_trend_ < 0.05) (Figure 2).

### 3.3. Influences of SNPs on Genetic Damage Levels

The basic information for 46 mirSNPs and 15 replaced SNPs are presented in Tables S1 and S2, respectively. The genotype distributions for 11 SNPs did not conform to the Hardy–Weinberg equilibrium (all *P*_HWE_ < 0.05), and thus were excluded from the following analysis. Rs73112689 were also excluded because all the subjects were of the same genotype (CC). The other 49 SNPs were distributed as expected under the Hardy–Weinberg equilibrium (all *P*_HWE_ > 0.05) (Tables S1 and S2), and were included in the following analysis.

**Figure 3:**
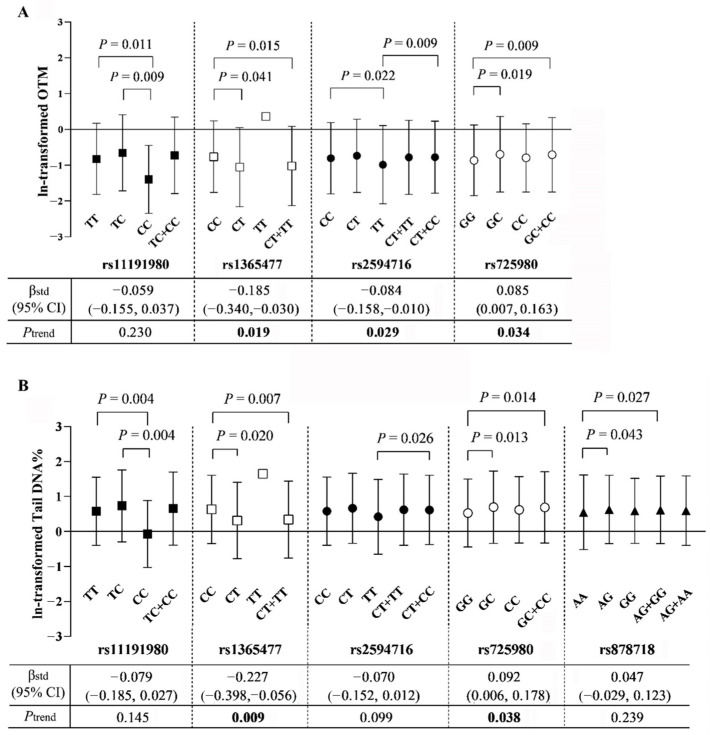
Significant effects of SNPs and/or their genotypes on (**A**) OTM and (**B**) tail DNA%. Abbreviations: OTM, olive tail moment; tail DNA%, percentage of DNA in the comet tail. The lines represent the levels of ln-transformed OTM or tail DNA% (error bars absent for condition where only two available samples). The between-genotype differences were evaluated by means of multivariate analysis of covariance, and the associations of the number of variant alleles with OTM and tail DNA% were evaluated by means of multivariate linear regression models with adjustment for age, gender, smoking status, pack-years of smoking, drinking status, factory location, and BMI. Bolded *p*-values indicate statistical significance.

We first compared the levels of OTM and tail DNA% in workers carrying different genotypes, and then investigated the associations of the number of variant alleles with OTM and tail DNA% (Table S6 and Figure 3). We found that after adjusting for multiple confounding factors, the genotypes and/or the numbers of mutant alleles of rs11191980, rs1365477, rs2594716, rs725980, and rs878718 significantly influenced OTM and/or tail DNA% (Table S6 and Figure 3). Specifically, for rs11191980, when compared with carriers of wild homozygotes (TT) and heterozygotes (TC), workers with mutant homozygotes (CC) had significantly lower mean OTM (TT: −0.82; TC: −0.65; CC: −1.40; *p* < 0.015) and tail DNA% (TT: 0.58; TC: 0.73; CC: −0.07; *p* = 0.004). For rs1365477, carriers of wild homozygotes (CC) had significant higher OTM (CC: −0.77; CT: −1.05; CT + TT: −1.02; *p* < 0.045) and tail DNA% (CC: 0.63; CT: 0.31; CT + TT: 0.34; *p* < 0.025) when compared with the carriers of CT or T alleles (i.e., CT + TT), and the number of mutant T alleles was negatively associated with both OTM (β = −0.185, *P*_trend_ = 0.019) and tail DNA% (β = −0.227, *P*_trend_ = 0.009). For rs2594716, when compared with carriers of wild homozygotes (CC) and/or C allele (i.e., CT + CC), subjects with mutant homozygotes (TT) had significantly lower mean OTM (CC: −0.80; CT + CC: −0.78; TT: −0.99; *p* < 0.025) and tail DNA% (CT + CC: 0.62; TT: 0.42; *p* = 0.026), and the number of mutant T alleles was significantly negatively associated with OTM (β = −0.084, *P*_trend_ = 0.029). For rs725980, when compared with carriers of wild homozygotes (GG), workers with GC or mutant C alleles (i.e., GC + CC) had significant higher OTM (GG: −0.87; GC: −0.70; GC + CC: −0.71; *p* < 0.020) and tail DNA% (GG: 0.53; GC: 0.70; GC + CC: 0.69; *p* < 0.035). Furthermore, the number of rs725980 C alleles was positively associated with OTM (β = 0.085, *P*_trend_ = 0.034) and tail DNA% (β = 0.092, *P*_trend_ = 0.038). For rs878718, when compared with the carriers of wild homozygotes (AA), workers with an AG genotype and mutant G alleles (i.e., AG + GG) had significant higher tail DNA% (AA: 0.55; AG: 0.63; AG + GG: 0.62; *p* < 0.045). However, we did not observe any statistically significant between-genotype differences (all *p* > 0.05) or associations (all *P*_trend_ > 0.05) in the two genetic damage indicators for other SNPs (Table S6).

### 3.4. Effect of Gene–Environment Interactions on Genetic Damage Levels

**Figure 4:**
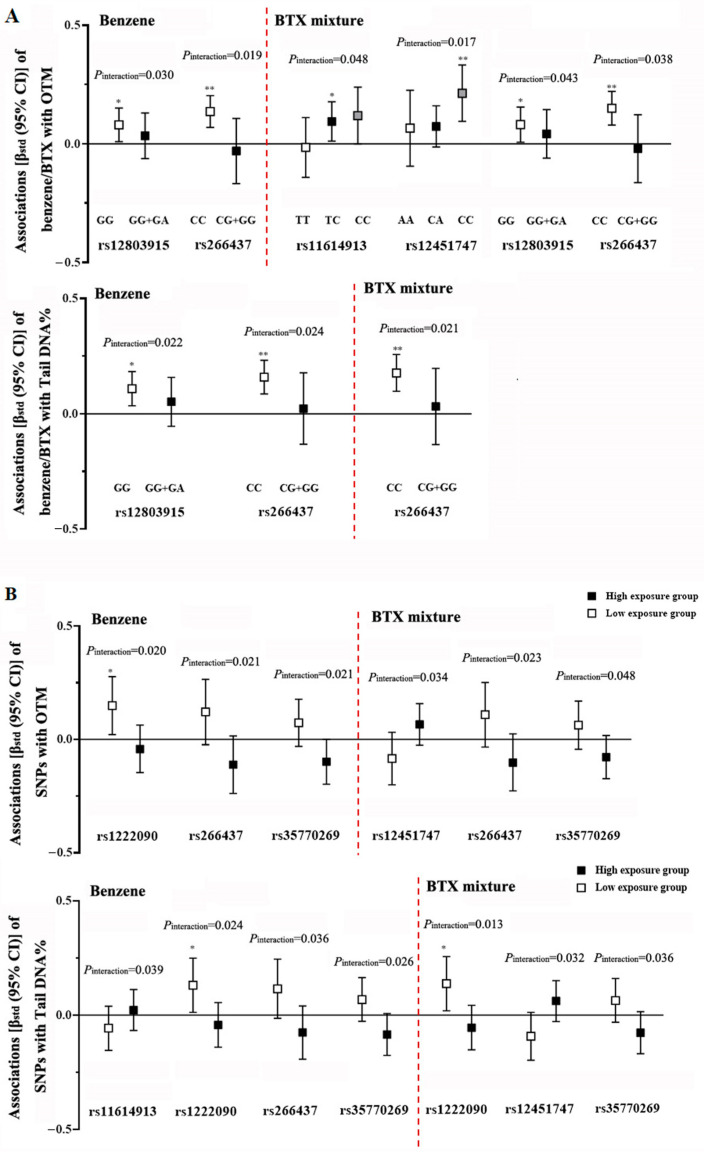
Significant modifying effects of (**A**) SNPs and (**B**) benzene/BTX exposure on each other’s associations with OTM and tail DNA%, with *P*_interaction_ < 0.05. Abbreviations: OTM, olive tail moment; Tail DNA%, percentage of DNA in the comet tail. The lines represent the associations [β_std_ (95% CI)] evaluated by multivariate linear regression models with adjustment for age, gender, smoking status, pack-years of smoking, drinking status, factory location, and BMI. * *P*_trend_ < 0.05, and ** *P*_trend_ < 0.01.

To explore the effect of gene–environment interactions of exposure to benzene and the BTX mixture with SNPs on genetic damage biomarkers, we evaluated their mutual effect modification on each other’s associations with OTM and tail DNA%. We observed that the positive associations of benzene and/or the BTX mixture with OTM and tail DNA% were significantly different among different genotypes of rs11614913, rs12451747, rs12803915, and/or rs266437, with more pronounced effect estimates (β_std_ ≥ 0.080, *P*_trend_ < 0.05) in carriers of rs11614913 TC heterozygotes and of wild homozygotes of rs12451747 (i.e., CC), rs12803915 (i.e., GG), and rs266437 (i.e., CC) (*P*_interaction_ ≤ 0.05) (Figure 4 and Table S7). However, SNPs which were found to be associated with genetic damage levels (including rs11191980, rs1365477, rs2594716, rs725980, and rs878718) had no modifying effects on the effect estimates of exposure to benzene and the BTX mixture (Table S7).

We then investigated the modifying effects of benzene and the BTX mixture on the associations of SNPs with genetic damage levels. We stratified the subjects included in mirSNP analysis into two exposure groups based on the median benzene CE levels and BTX wqs index. We observed that the effect estimates of rs11614913, rs12220909, rs12451747, rs266437, and/or rs35770269 on OTM and/or tail DNA% were significantly different in workers with different exposure levels of benzene and/or the BTX mixture (*P*_interaction_ < 0.05) (Figure 4 and Table S8). Interestingly, the positive associations of rs12220909 with OTM and/or tail DNA% were more pronounced (β_std_ > 0.130, *P*_trend_ ≤ 0.030) in workers with lower exposure levels of benzene and BTX mixture, while they were weaker in workers with higher exposure levels (β_std_ < −0.040, *P*_trend_ > 0.05). However, exposure to benzene and the BTX mixture had no modifying effects on the effect estimates of SNPs that were associated with genetic damage levels (Table S8).

## 4. Discussion

To the best of our knowledge, the present study was the first exploration into the effects of BTX exposure, along with polymorphisms in miRNA genes, and the gene–environment interactions on genetic damage in Chinese petrochemical workers. We found that exposure to benzene and the BTX mixture might induce a significant increase in DNA strand break levels. Interestingly, the effects of benzene were significantly enhanced by higher exposure levels of toluene and xylene. Furthermore, these effects of benzene and BTX mixture were more pronounced in carriers of rs11614913 heterozygotes and of wild homozygotes of rs12451747, rs12803915, and rs26643. In addition, there were significant negative associations of rs1365477 and rs2594716, and positive associations of rs725980, with DNA strand break levels in the total population. Furthermore, the positive associations of rs12220909 with DNA strand break levels were more pronounced in workers with lower exposure levels to benzene and the BTX mixture. Our findings provide further support for the involvement of BTX co-exposure, mirSNPs, and their gene–environment interactions in determining the severity of genetic damage in a complex manner.

In epidemiological studies of BTX components, especially at low exposure levels, quality of exposure assessment is closely related to the ability to detect their health risks. Internal exposure biomarkers have a short biological resident time and are very susceptible to confounding factors such as smoking and diet [25], making them less suitable for long-term low-dose exposure assessment. Given that inhalation is the most common exposure route for volatile BTX components, and that the TWA concentrations of BTX components in our participants’ workplaces were relatively stable and considerably lower than their commonly recommended occupational exposure limit [10], we used long-term environment monitoring data and work years to derive individual cumulative exposure estimates.

Our findings are consistent with previous studies showing that low-dose benzene exposure is associated with genetic damage. Several recent comprehensive meta-studies have indicated that compared to the control group, benzene exposure, even lower than 3.25 mg/m^3^, might induce significant increases in various genetic damage indictors in blood lymphocytes [12,15]. A study involving workers with benzene cumulative doses ranging from 1.19 to 20.87 mg/m^3^ × year showed a significant association between benzene exposure dose and PIG-A mutant frequencies and MN frequencies [26]. Li et al. suggested that low occupational benzene exposure ranging from 0.33 mg/m^3^ to 1.08 mg/m^3^ could induce a significantly increased MN frequency in lymphocytes [27]. The median CE levels and 8 h-TWA concentrations of benzene for our subjects were 0.66 mg/m^3^ × year and 0.036 mg/m^3^, respectively, which were lower than the levels in the above-mentioned studies. Even at such low exposure levels, the present study still observed significant associations of benzene CE levels with OTM and tail DNA%, further proving that long-term exposure to even lower benzene levels could still induce a significant increase in DNA strand break levels. We further found that the effect of a low dose of benzene on DNA strand break was more pronounced in subjects older than 40 years. Age is an important biological factor influencing susceptibility to the toxic effects of environmental hazards by affecting the efficiency of the pathways involved in metabolism and cytotoxic outcomes [28]. Gaikwad et al. [29] found that compared to younger subjects, older ones were more susceptible to oxidative DNA damage caused by exposure to genotoxic substances. Furthermore, older workers were exposed to BTX for extended periods, which may lead to the accumulation of more genetic damage.

BTX components frequently co-exist in various human environments. Recent studies suggest caution in interpreting the health effects of mixed environmental exposures due to complex interactions among multiple components [30]. To address the complexities of mixed BTX exposures in real-world settings, we employed both single-pollutant models and combined exposure models to better understand each compound’s toxicological role. We adopted both gWQS models and interaction analysis in the present study to explore the combined effects of three BTX components on genetic damage indices. Although the individual effects of toluene and xylene were not significant and their contributions to the combined BTX effects were limited, interaction analysis revealed that benzene’s genotoxicity was more pronounced in individuals with moderate to high toluene and xylene exposure levels and was significantly increased with higher xylene exposure. Simultaneous exposure to BTX compounds may alter the metabolism mechanisms and further influence the toxicity of each component [8]. Earlier studies addressing co-exposure to benzene and toluene at high concentrations demonstrated a reduction in benzene-induced genotoxicity, which may be related to the dose-dependent competitive inhibition of metabolism [31]. Conversely, another study indicated that at lower exposure levels (benzene at 50 ppm and toluene at 100 ppm), the induction of CYP2E1 by toluene could lead to the increased metabolism of benzene into its genotoxic metabolite hydroquinone, potentially causing greater genetic damage [9]. In the context of the frequent low-dose co-existence of BTX compounds in human environments, our findings, along with those of previous studies, suggest that the genetic damage induced by benzene at low doses may be influenced by concurrent exposure to toluene and xylene. However, it is important to note that these interactions at low doses may involve distinct metabolic pathways compared to those at higher doses. Therefore, our study highlights the need for comprehensive management strategies that consider not only the levels of individual compounds like benzene but also the potential interactions at low doses of toluene and xylene.

In addition to the influence of BTX components, individually and in various combinations, on genetic damage, we also evaluated the involvement of genetic polymorphisms on miRNA genes. We observed the significant effects of the genotypes and/or the numbers of mutant alleles of rs11191980, rs1365477, rs2594716, rs725980, and rs878718 on DNA strand break levels. Rs11191980 is in high linkage disequilibrium with rs45596840 situated within the seed sequence of miR-4482. Mumtaz et al. found that the expression of mir-4482 was decreased in prostate cancer tissues when compared with the adjacent normal tissues and could inhibit the progression of prostate cancer cells by suppressing the expression of ETS-related gene [32]. Rs7259810 is in high linkage disequilibrium with rs2241347 in the mature miR-3130 sequence. Mir-3130 has been found to inhibit the expression of NDUFS1 to promote the invasiveness of lung adenocarcinoma in vivo and in vitro [33] and could also regulate the miR-3130-3p/NFYA/SATB1 axis to promote the occurrence and development of endometrial cancer cells [34]. Rs1365477 is in high linkage disequilibrium with rs12473206 located in the mature region of miR-4433. Wu et al. identified that mir-4433 could induce apoptosis through targeting Bcr-Abl genes and suppress the growth of leukemia cells [35]. Rs2594716 is closely linked to rs895819, which is positioned within the loop of the pre-miR-27a sequence. Previous studies have indicated a significant association between rs895819 and tumor susceptibility, with carriers of the mutant allele demonstrating a notably reduced risk of diffuse large B-cell lymphoma compared with individuals possessing the wild-type genotype [36]. Our findings also suggest that the population carrying the mutant allele at this specific locus exhibited lower genetic damage levels, suggesting that the mutant allele of rs2594716 (or rs895819) might exhibit a protective effect against cancer risk by reducing genetic damage. Rs878718 is tightly linked to rs5997893 located in the pre-miR-3928 sequence. Mir-3928 has been identified as a tumor-suppressive miRNA in both in vivo and in vitro, and is capable of downregulating oncogenes and upregulating p53, thereby inhibiting the progression of glioblastoma [37]. Collectively, our findings suggest potential roles for these SNPs in genetic damage, which could lead to differential cancer susceptibility. However, the mechanisms by which these SNPs influence miRNA biogenesis, activity, and/or bioavailability remain to be elucidated by further investigations.

In order to explore the gene–environment interactions, we further assessed the mutual modifying influence of SNPs and environment factors (including benzene and the BTX mixture) on each other’s associations with genetic damage. The effects of benzene and the BTX mixture were found to be more pronounced among carriers of rs11614913 heterozygotes and wild-type homozygotes of rs12451747, rs12803915, and rs266437. Rs11614913 is a well-studied SNP located within the mature miRNA-196a2 sequence. Its mutant allele has been implicated in elevating the miRNA expression levels and enhancing the regulatory capacity of multiple target genes [38], eventually increasing the risk of cancers like acute lymphoblastic leukemia (ALL) in Chinese children [39]. The present study also found that individuals with the rs11614913 mutant allele might experience more severe DNA strand breaks as a result of exposure to benzene and the BTX mixture. However, our previous research observed that the rs11614913 mutant allele might attenuate the effects of lead, naphthalene, and benzo[a]pyrene on increased oxidative DNA damage among coke oven workers [21]. These seemingly contradictory results might be explained by the different balance of the functions of miR-196a2-targeting genes (including oncogenes or tumor suppression genes) in specific exposure conditions [40]. However, the regulation mechanisms of rs11614913 warrant further investigation. Rs12803915 is located in the mir-612 precursor coding region and has been shown to be significantly associated with ALL risk in a case–control study, with the mutant allele being protective [41]. It has been shown that rs12803915 could significantly influence the expression levels of mature miR-612 [42], which may further regulate the downstream targets and thereby affect the susceptibility to toxic effects of environmental hazards like benzene. Rs12451747 is located in the mature sequence of miR-1296b, which has been documented to be highly expressed in various cancers [43] and may be involved in the activation of the PI3K/AKT signaling pathway [44]. Rs266437 is linked to rs266435 in the hsa-mir-4804 precursor coding region, yet reports on the links of these loci and the associated miRNA with cancers are scarce. Overall, the present study might extend these findings by revealing the interactive effects of these SNPs with benzene/BTX exposure and suggesting that individuals with specific alleles at these loci may experience increased genetic damage subsequent to benzene/BTX exposure, potentially heightening the cancer risk. Furthermore, our study also observed a significant association of rs12220909 with higher genetic damage levels in the low-exposure group of benzene and/or the BTX mixture, which became weaker and insignificant in the high-exposure group. Rs12220909, situated in the seed sequence of mir-4293, has been implicated in tumorigenesis, with its heterozygotes often being regarded as a protective factor against cancers [45]. Our findings further suggest that the protective capacity of rs12220909 against tumorigenesis may be dose-dependent: the mutant allele might confer a protective advantage at higher exposures to benzene and BTX mixture, and may not provide the same levels of protection at lower exposures, potentially leading to increased genetic damage susceptibility.

The present study has several strengths. Firstly, we calculated individual BTX CE levels based on long-term average TWA concentrations and working years, thus providing a representation of environmental exposure profiles of petrochemical workers subjected to low BTX concentrations. Additionally, we performed an integrative bioinformatics analysis based on a solid public database and an extensive literature review to screen out multiple functional miRNA-related SNPs, which might help to systematically evaluate the effects of miRNA-related SNPs. However, there also existed some limitations in our study. First, our study is an exploratory cross-sectional study, and thus the findings could not demonstrate the causal associations. Additionally, although we selected petrochemical workers from workshops where BTX were the primary occupational hazards, they may still be exposed to other genotoxic hazards in their working and living environment, causing challenges in describing the genotoxicity of BTX in environments exposed to complex contaminants. Although we adjusted for general characteristics and factory location in our statistical analysis to minimize their confounding effects, further studies are needed to evaluate their influences on our results. Furthermore, the present study was limited to an ethnic Chinese occupational population, and it is uncertain whether our findings can be extrapolated to other populations. Additionally, in the subsequent studies, some research subjects were not included in the mir-SNP analysis due to limited biological samples, necessitating further examination of the representativeness of the results.

## 5. Conclusions

Conclusively, our study reveals that low-dose benzene exposure, particularly within BTX mixtures, significantly contributes to DNA strand breaks. The genotoxic effects of benzene are amplified by higher levels of toluene and xylene co-exposure. Furthermore, individuals harboring rs11614913 heterozygotes and wild homozygotes of rs12451747, rs12803915, and rs266437 appeared to be more vulnerable to the genotoxic effects of benzene and the BTX mixture. Moreover, the genotypes and/or the number of mutant alleles of rs11191980, rs1365477, rs2594716, rs725980, and rs878718 might influence DNA strand break levels. We also observed more pronounced positive associations of rs12220909 with DNA strand break levels only in workers with lower exposure levels of benzene and BTX mixture. These findings highlight the complex interactions between benzene exposure, mirSNPs, and genetic damage, emphasizing the need for further research to inform targeted interventions and refine risk assessment strategies for BTX-exposed workers. The present study contributes some novel insights into the individual and interactive effects of BTX and miRNA-related SNP on genetic damage, further shedding light on the risk assessment of genetic damage in BTX-exposed occupational populations.

## Figures and Tables

**Table 1:** General characteristics, BTX exposure levels, and genetic damage levels in petrochemical workers (n = 1083).

Variables	Mean ± SD, n (%), or Median (25th Percentile, 75th Percentile)
General characteristics	
Age (years)	40.0 ± 7.0
Gender [male/female (%male)]	798/285 (73.68)
Smoking status [smoker/nonsmoker (%smoker)]	338/745 (31.21)
Pack-years of smoking	2.8 ± 6.2
Drinking status [drinker/nondrinker (%drinker)]	492/591 (45.43)
Factory location [Maoming/Guangzhou (%Maoming)]	707/376 (65.28)
Working years (years)	19.2 ± 7.8
BMI (kg/m^2^)	23.2 ± 3.0
BTX CE levels (mg/m^3^ × year)	
Benzene	0.66 (0.37, 1.00)
Toluene	0.78 (0.50, 1.21)
Xylene	1.46 (0.61, 2.50)
Genetic damage indices	
MN frequency (per 1000 cells)	9 (6, 13)
NPB frequency (per 1000 cells)	1 (0, 2)
NBUD frequency (per 1000 cells)	2 (1, 3)
OTM	0.35 (0.20, 1.18)
Tail DNA%	1. 50 (0.86, 4.83)
Tail moment	0.13 (0.05, 0.69)

Abbreviations: BTX, benzene, toluene, and xylene; BMI, body mass index; CE, cumulative exposure; MN, micronucleus; NPB, nucleoplasmic bridge; NBUD, nuclear bud; OTM, olive tail moment; Tail DNA%, percentage of DNA in the comet tail.

**Table 2:** Associations of single BTX CE levels with genetic damage indices.

BTX Components	MN Frequency	NPB Frequency	NBUD Frequency	OTM	Tail DNA%	Tail Moment
Benzene						
High group ^a^	10.26 ± 5.87	1.39 ± 1.76	2.63 ± 2.87	1.31 ± 1.00	4.98 ± 3.42	0.91 ± 1.12
Low group ^a^	9.20 ± 5.29	1.38 ± 1.48	1.85 ± 1.77	0.50 ± 0.76	2.02 ± 2.57 *	0.29 ± 0.83
β_std_ (95% CI) ^b^	−0.057 (−0.142, 0.029)	−0.032 (−0.122, 0.057)	−0.015 (−0.102, 0.073)	0.048 (0.007, 0.090)	0.073 (0.027, 0.119)	0.041 (−0.001, 0.084)
*P*_trend_^b^	0.193	0.480	0.742	0.022	0.002	0.057
Toluene						
High group ^a^	9.71 ± 5.66	1.38 ± 1.60	1.39 ± 1.66	0.79 ± 0.97	3.07 ± 3.29	0.52 ± 1.07
Low group ^a^	9.71 ± 5.55	1.39 ± 1.66	2.31 ± 2.71	0.75 ± 0.89	2.96 ± 3.12 *	0.47 ± 0.89
β_std_ (95% CI) ^b^	0.051 (−0.132, 0.030)	−0.053 (−0.136, 0.030)	0.020 (−0.062, 0.102)	0.023 (−0.019, 0.066)	0.034 (−0.012, 0.081)	0.014 (−0.029, 0.057)
*P*_trend_^b^	0.219	0.211	0.634	0.276	0.150	0.533
Xylene						
High group ^a^	9.22 ± 5.68	1.33 ± 1.40	1.63 ± 1.75	0.42 ± 0.69	1.72 ± 2.25	0.23 ± 0.79
Low group ^a^	10.06 ± 5.53	1.42 ± 1.76	2.64 ± 2.70	1.22 ± 1.00	4.68 ± 3.47	0.84 ± 1.09
β_std_ (95% CI) ^b^	−0.072 (−0.170, 0.025)	−0.050 (−0.152, 0.051)	−0.019 (−0.118, 0.080)	−0.004 (−0.054, 0.045)	0.001 (−0.054, 0.056)	−0.020 (−0.071, 0.031)
*P*_trend_^b^	0.144	0.331	0.704	0.864	0.962	0.435

Abbreviations: BTX, benzene, toluene, and xylene; CE, cumulative exposure; MN, micronucleus; NPB, nucleoplasmic bridge; NBUD, nuclear bud; OTM, olive tail moment; Tail DNA%, percentage of DNA in the comet tail. ^a^: The mean ± SD for each genetic damage indicator. ^b^: Generalized linear models (GLM) with adjustment for age, gender, smoking status, pack-years of smoking, drinking status, factory location, and BMI. BTX CE were included as a continuous variable in the GLM. *: *p* < 0.05 for the between-group differences which were determined by multivariable covariance analysis with adjustment for age, gender, smoking status, pack-years of smoking, drinking status, factory location, and BMI.

## Data Availability

The data presented in this study are available upon request from the corresponding author.

## Supplementary Materials

The following supporting information can be downloaded at: https://www.mdpi.com/article/10.3390/toxics12110821/s1, Table S1: Basic information of the mirSNPs included in the present study. Table S2: Basic information on the replaced SNPs for mirSNPs which failed in primer design in the present study. Table S3: Comparisons of genetic damage levels in subjects with different general characteristics. Table S4: General characteristics, BTX exposure levels, and genetic damage levels in workers that were or were not included in mirSNP study. Table S5: Associations of benzene CE levels with OTM and Tail DNA% in workers with different general characteristics. Table S6: Effects of SNPs and their genotypes on the levels of OTM and Tail DNA%. Table S7: Modifying effects of SNPs on the associations [βstd (95% CI)] of benzene and BTX mixture with OTM and Tail DNA%. Table S8: Modifying effects of benzene exposure on the associations [βstd (95% CI)] of SNPs (continuous) with OTM and Tail DNA%. Figure S1: Associations of BTX CE levels with OTM and Tail DNA% in subjects included in mirSNP analysis (n = 667).
