# Supplementary material for: Effects of Co-Exposure to Benzene, Toluene, and Xylene, Polymorphisms of microRNA Genes, and Their Interactions on Genetic Damage in Chinese Petrochemical Workers
Source: Toxics. 2024 Nov 16;12(11):821. doi: 10.3390/toxics12110821 (PMC11598265; doi:10.3390/toxics12110821)
Supplement: Supplementary file 1 [file toxics-12-00821-s001.zip › toxics-3227570-supplementary.pdf]

Supplemental Material

BTX exposure, microRNA genes single nucleotide polymorphisms and their interaction on genetic damage levels in Chinese petrochemical workers

Affiliations:

Table of Contents	Page
**Table S1** Basic information of the mirSNPs included in the present study.	2
**Table S2** Basic information of the replaced SNPs for mirSNPs which were failed in primer design in the present study	4
**Table S3** Comparisons of genetic damage levels in subjects with different general characteristics.	5
**Table S4** General characteristics, BTX exposure levels, and genetic damage levels in workers that were or were not included in mirSNP study.	6
**Table S5** Associations of benzene CE levels with OTM and Tail DNA% in workers with different general characteristics.	7
**Table S6** Effects of SNPs and their genotypes on the levels of OTM and Tail DNA%.	8
**Table S7** Modifying effects of SNPs on the associations [βstd (95% CI)] of benzene and BTX mixture with OTM and Tail DNA%.	13
**Table S8** Modifying effects of benzene exposure on the associations [βstd (95% CI)] of SNPs (continuous) with OTM and Tail DNA%.	18
**Figure S1** Associations of BTX CE levels with OTM and Tail DNA% in subjects included in mirSNP analysis (n=667)	22

Table S1. Basic information of the mirSNPs included in the present study.

mirSNP	microRNA	Functional coding region	Chromosome	Allele (A/a*)	Genotype (AA/Aa/aa)	Call rate (%)	P_{HWE} [a]
rs10061133	miR-449b	Mature	5	A/G	352/278/31	99.10	**0.010**
rs11237828	miR-5579	Mature	11	T/C	220/328/108	98.35	0.442
rs11614913	miR-196a-2	Mature	12	T/C	200/343/113	98.35	0.099
rs12220909	miR-4293	Seed sequence	10	G/C	432/201/33	99.85	0.131
rs12355840	miR-202	Pre-miRNA	10	T/C	432/81/4	77.51	0.924
rs12402181	miR-3117	Seed sequence	1	G/A	325/276/62	99.40	0.758
rs12451747	miR-1269b	Mature	17	A/C	140/262/106	76.16	0.415
rs12803915	miR-612	Pre-miRNA	11	G/A	469/183/10	99.25	0.096
rs13299349	miR-3152	Mature	9	G/A	532/119/10	99.10	0.266
rs2043556	miR-605	Pre-miRNA	10	T/C	385/238/38	99.10	0.879
rs2070960	miR-3620	Seed sequence	1	C/T	429/206/29	99.55	0.501
rs2114358	miR-1206	Pre-miRNA	8	A/G	307/291/61	98.80	0.502
rs2168518	miR-4513	Seed sequence	15	G/A	445/191/29	99.70	0.147
rs2292832	miR-149	Pre-miRNA	2	T/C	208/242/59	76.31	0.367
rs231027	miR-4731	Pre-miRNA	17	T/C	156/256/101	76.91	0.827
rs2620381	miR-627	Seed sequence	15	A/C	563/85/5	97.90	0.370
rs2910164	miR-146a	Seed sequence	5	C/G	245/321/87	97.90	0.258
rs34115976	miR-577	Pre-miRNA	4	C/G	592/71/3	99.85	0.582
rs35770269	miR-449c	Seed sequence	5	A/T	324/272/65	99.10	0.476
rs3823658	miR-5090	Seed sequence	7	G/A	503/142/15	98.95	0.193
rs4919510	miR-608	Mature	10	G/C	200/329/133	99.25	0.913
rs55680837	miR-3622b	Pre-miRNA	8	G/C	163/252/98	76.91	0.973
rs56103835	miR-323b	Pre-miRNA	14	C/T	225/223/68	77.36	0.281

rs56863230	miR-1208	Pre-miRNA	8	G/C	464/48/2	77.06	0.529
rs60432575	miR-4532	Seed sequence	20	G/A	580/82/5	100	0.270
rs60871950	miR-4467	Mature	7	G/A	397/220/36	97.90	0.449
rs61992671	miR-412	Mature	14	A/G	598/65/3	99.85	0.395
rs6513497	miR-646	Mature	20	T/G	539/97/9	96.70	0.060
rs67106263	miR-3144	Mature	6	G/A	505/148/9	99.25	0.618
rs73239138	miR-1269a	Mature	4	G/A	296/291/66	97.90	0.655
rs73410309	miR-4739	Mature	17	G/C	575/89/2	99.85	0.457
rs75330474	miR-323b	Mature	14	C/T	593/67/2	99.25	0.941
rs77639117	miR-576	Pre-miRNA	4	A/T	478/39/1	77.66	0.827
rs79402775	miR-933	Mature	2	G/A	477/165/15	98.50	0.870
rs174561	miR-1908	Pre-miRNA	11	C/T	164/281/58	75.41	**<0.001**
rs2292181	miR-564	Pre-miRNA	3	G/C	322/51/44	62.52	**<0.001**
rs2296319	miR-4706	Mature	14	G/A	506/10/145	99.10	**<0.001**
rs4414449	miR-548ap	Mature	15	A/G	565/6/77	97.15	**<0.001**
rs515924	miR-548al	Seed sequence	11	A/G	352/199/114	99.70	**<0.001**
rs5965660	miR-888	Pre-miRNA	X	T/G	493/5/22	77.96	**<0.001**
rs7210937	miR-1269b	Seed sequence	17	G/C	230/324/112	99.85	0.907
rs78212770	miR-629	Mature	15	C/G	416/0/238	98.05	**<0.001**
rs78861479	miR-516b1	Mature	19	G/A	462/10/0	70.76	0.816
rs79759099	miR-1304	Seed sequence	11	A/G	4/661/0	99.70	**<0.001**
rs79966586	miR-585	Pre-miRNA	5	C/T	277/95/39	61.62	**<0.001**
rs73112689	miR-4459	Mature	5	C/T	666/0/0 666	99.85	**-**

* A: Wild-type genotype; a: Mutant genotype;

[a] P_{HWE}: P values for Hardy-Weinberg equilibrium by goodness-of-fit Chi-square test.

Table S2. Basic information of the replaced SNPs for mirSNPs which were failed in primer design in the present study.

Replaced SNP	Original mirSNPs					Allele (A/a*)	Genotype (AA/Aa/aa)	Call rate (%)	P_{HWE} [a]
	mirSNP	microRNA	Functional coding region	Chromosome	r^2				
rs11191980	rs45596840	miR-4482	Seed sequence	10	1.000	T/C	388/115/12	77.21	0.323
rs112250173	rs12314280	miR-5700	Mature	12	1.000	T/G	403/106/8	77.51	0.734
rs1365477	rs12473206	miR-4433	Mature	2	0.960	C/T	460/49/2	76.61	0.573
rs1427865	rs2042253	miR-5197	Pre-miRNA	5	0.870	G/C	183/250/84	77.51	0.929
rs218662	rs8078913	miR-4520a	Mature	17	0.817	A/G	280/198/41	77.81	0.471
rs2594716	rs895819	miR-27a	Pre-miRNA	19	0.967	C/T	279/195/41	77.21	0.400
rs266437	rs266435	miR-4804	Seed sequence	5	0.857	C/G	366/133/16	77.21	0.362
rs2967615	rs72996752	miR-4999	Mature	19	0.957	C/T	258/213/43	77.06	0.918
rs303064	rs9295535	miR-5689	Mature	6	1.000	C/T	247/217/49	76.91	0.894
rs3746448	rs3746444	miR-499	Seed sequence	20	1.000	C/T	310/284/23	92.50	**<0.001**
rs4294864	rs6505162	miR-423	Pre-miRNA	17	0.955	C/G	310/184/23	77.51	0.512
rs725980	rs2241347	miR-3130	Mature	2	0.964	G/C	286/204/28	77.66	0.280
rs7954025	rs61938575	miR-3922	Mature	12	0.980	A/G	286/188/40	77.06	0.246
rs8016027	rs2273626	miR-4707	Seed sequence	14	0.959	C/T	319/170/27	77.36	0.486
rs878718	rs5997893	miR-3928	Pre-miRNA	22	0.986	A/G	154/269/92	77.21	0.173

* A: Wild-type genotype; a: Mutant genotype;

[a] P_{HWE}: *P* values for Hardy-Weinberg equilibrium by goodness-of-fit Chi-square test.

Table S3. Comparisons of genetic damage levels in subjects with different general characteristics [a].

Genetic damage indices [b]	Age		Gender		Smoking status		Drinking status		BMI	
	≤40 years (n = 590)	>40 years (n = 493)	Female (n = 285)	Male (n = 798)	Non-smoker (n = 745)	Smoker (n = 338)	Non-drinker (n = 591)	Drinker (n = 492)	<24 kg/m^2 (n = 680)	≥24 kg/m^2 (n = 403)
MN frequency	9.41±5.41	10.17±5.87	**10.68±6.08**	**9.33±5.36***	9.65±5.58	9.85±5.66	9.96±5.72	9.41±5.46	9.39±5.50	10.27±5.75
NPB frequency	1.36±1.68	1.43±1.55	1.27±1.39	1.43±1.71	1.36±1.61	1.43±1.67	1.35±1.62	1.42±1.64	1.41±1.70	1.34±1.05
NBUD frequency	2.14±2.15	2.39±2.77	2.51±2.98	2.13±2.14	2.22±2.43	2.28±2.40	2.31±2.55	2.15±2.26	2.19±2.53	2.32±2.20
OTM [c]	-0.64±1.02	-0.97±0.97	**-0.71±1.05**	**-0.82±1.00***	-0.80±1.00	-0.77±1.06	-0.81±1.01	-0.77±1.02	-0.79±1.04	-0.80±0.98
Tail DNA% [c]	0.78±0.99	0.45±0.94	0.71±1.00	0.60±0.97	0.63±0.96	0.63±1.02	0.60±0.98	0.65±0.98	0.63±1.00	0.62±0.94
Tail moment [c]	-1.58±1.50	-2.08±1.45	**-1.69±1.54**	**-1.85±1.48***	-1.83±1.47	-1.77±1.56	-1.85±1.48	-1.76±1.51	-1.81±1.53	-1.81±1.44

Note: Abbreviations: BMI, body mass index; MN, micronucleus; NPB, nucleoplasmic bridge; NBUD, nuclear bud; OTM, Olive tail moment; Tail DNA%, percent DNA in the Comet tail.

[a] The between-group differences were determined by multivariable covariance analysis, with adjustment for age, gender, smoking status, pack-years of smoking, drinking status, factory location, and/or BMI when appropriate.

[b] Values of variables were shown by mean ± SD.

[c] Ln-transformed.

* $P < 0.05$.

Table S4. General characteristics, BTX exposure levels, and genetic damage levels in workers that were or were not included in mirSNP study.

Variables [a]	Subjects included in mirSNP analysis (n = 667)	Subjects not included in mirSNP analysis (n = 416)	*P* value
**General characteristics**			
Age (years)	40.38±7.05	39.57±6.80	0.063 [b]
Gender [male/female (%male)]	525/142 (78.7)	273/143(65.6)	**<0.001** [b]
Smoking status [smoker/nonsmoker (%smoker)]	245/422 (36.7)	94/322 (22.6)	**<0.001** [b]
Pack-years of smoking	3.29±6.75	2.02±5.25	**0.001** [b]
Drinking status [drinker/nondrinker (%drinker)]	317/350 (47.5)	178/238 (42.8)	0.128[b]
Factory location [Maoming/Guangzhou (%Maoming)]	475/192 (71.2)	238/178 (57.2)	**<0.001** [b]
Working years (years)	19.68±7.95	18.62±7.48	**0.028** [b]
BMI (kg/m^2)	23.35±3.18	22.98±2.81	**0.047** [b]
**BTX CE levels (mg/m^3 × year)**			
Benzene	0.58 (0.35, 0.99)	0.74 (0.47, 0.99)	0.724 [c]
Toluene	0.77 (0.53, 1.17)	0.80 (0.50, 1.32)	0.823 [c]
Xylene	1.57 (0.76, 2.43)	1.41 (0.38, 2.74)	0.690 [c]
**Genetic damage indices**			
MN frequency (per 1000 cells)	10 (6, 12)	9 (5, 13)	0.077 [c]
NPB frequency (per 1000 cells)	1 (0, 2)	1 (0, 2)	0.224[c]
NBUD frequency (per 1000 cells)	2 (1, 3)	2 (1, 3)	0.592 [c]
OTM	0.32 (0.20, 0.92)	0.46(0.21, 1.41)	0.465 [c]
Tail DNA%	1.33 (0.81, 3.71)	2.04(0.93, 5.49)	0.210[c]
Tail moment	0.11 (0.05, 0.48)	0.17 (0.05, 0.85)	0.634 [c]

Note: Abbreviations: BTX, benzene, toluene, and xylene; CE, cumulative exposure; MN, micronucleus; NPB, nucleoplasmic bridge; NBUD, nuclear bud; OTM, Olive tail moment; Tail DNA%, percent DNA in the Comet tail.

[a] Mean±SD, n (%), or median (P25, P75).

[b] Student's *t*-test for continuous variables and Chi-square test for categorical variables.

[c] Multivariable covariance analysis with adjustment for age, gender, smoking status, pack-years. of smoking, drinking status, factory location, and BMI.

Table S5. Associations of benzene CE levels with OTM and Tail DNA% in workers with different general characteristics.

General characteristics	Associations of benzene CE levels [a] with OTM [a]			Associations of benzene CE levels [a] with Tail DNA% [a]		
	β_{std} (95% CI)	P_{trend} [b]	$P_{interaction}$ [c]	β_{std} (95% CI)	P_{trend} [b]	$P_{interaction}$ [c]
**Age**						
≤40 (n = 590)	0.018 (-0.021, 0.057)	0.375	**3.79×10^{-4}**	0.030 (-0.015, 0.075)	0.190	**0.001**
>40 (n = 493)	0.175 (0.089, 0.261)	6.86×10^{-5}		0.201 (0.107, 0.295)	3.743×10^{-5}	
**Gender**						
Female (n = 285)	-0.001 (-0.095, 0.093)	0.985	0.478	0.030 (-0.068, 0.128)	0.556	0.862
Male (n = 798)	0.065 (0.018, 0.112)	0.008		0.094 (0.041, 0.147)	0.001	
**Smoking status**						
Non-smoker (n = 745)	0.039 (-0.010, 0.088)	0.118	0.399	0.055 (0.002, 0.108)	0.042	0.552
Smoker (n = 338)	0.065 (-0.019, 0.149)	0.131		0.122 (0.026, 0.218)	0.013	
**Drinking status**						
Non-drinker (n = 591)	0.042 (-0.017, 0.101)	0.158	0.981	0.067 (0.002, 0.132)	0.041	0.585
Drinker (n = 492)	0.063 (-2.61×10^{-4}, 0.126)	0.046		0.097 (0.028, 0.166)	0.006	
**BMI**						
<24 kg/m^2 (n = 680)	0.035 (-0.014, 0.084)	0.169	0.248	0.047 (-0.006, 0.100)	0.083	0.171
≥24 kg/m^2 (n = 403)	0.071 (-0.005, 0.147)	0.068		0.113 (0.027, 0.199)	0.010	

[a] Z-Score standardization after ln-transformed.

[b] Generalized linear models with adjustment for age, gender, smoking status, pack-years of smoking, drinking status, factory location, and/or BMI when appropriate.

[c] $P_{interaction}$ was calculated by entering an interaction term of benzene CE levels (continuous) and general characteristics (categorical) into generalized linear models with adjustment for age, gender, smoking status, pack-years of smoking, drinking status, factory location, and/or BMI when appropriate.

Table S6. Effects of SNPs and their genotypes on the levels of OTM and Tail DNA%.

SNP	Genotype	N	OTM (mean ± SD) [a]	Associations of SNPs with OTM[b]		Tail DNA% (mean ± SD) [a]	Associations of SNPs with Tail DNA%[b]	
				β_{std} (95%CI)	P_{trend} [c]		β_{std} (95%CI)	P_{trend} [c]
rs11191980	TT	388	-0.82±0.99	-0.059 (-0.155, 0.037)	0.230	0.58±0.98	-0.079 (-0.185, 0.027)	0.145
	TC	115	**-0.65±1.06**[#]			**0.73±1.03**[#]		
	CC	12	**-1.40±0.95**[*]			**-0.07±0.96**[*]		
	TC+CC	127	-0.73±1.07			0.66±1.05		
rs112250173	TT	403	-0.80±1.02	0.080 (-0.024, 0.184)	0.134	0.60±0.99	0.049 (-0.065, 0.163)	0.403
	TG	106	-0.75±1.01			0.62±1.01		
	GG	8	-0.91±0.97			0.44±0.91		
	TG+GG	114	-0.76±1.01			0.61±1.00		
rs11237828	TT	230	-0.89±1.00	0.019 (-0.048, 0.086)	0.583	0.52±0.98	0.018 (-0.053, 0.089)	0.620
	TC	328	-0.89±0.99			0.51±0.97		
	CC	108	-0.94±0.98			0.48±0.96		
	TC+CC	436	-0.90±0.99			0.51±0.96		
	TT+TC	558	-0.89±0.99			0.52±0.97		
rs11614913	TT	200	-0.87±1.04	-0.021 (-0.090, 0.048)	0.545	0.55±1.02	-0.038 (-0.111, 0.035)	0.312
	TC	343	-0.91±0.95			0.51±0.92		
	CC	113	-0.87±1.04			0.50±1.02		
	TC+CC	456	-0.90±0.97			0.51±0.95		
	TT+TC	543	-0.89±0.98			0.52±0.96		
rs12220909	GG	432	-0.94±1.00	0.044 (-0.034, 0.122)	0.279	0.47±0.99	0.054 (-0.032, 0.140)	0.221
	GC	201	-0.85±0.95			0.56±0.92		
	CC	33	-0.60±0.99			0.82±0.88		
	GC+CC	234	-0.82±0.96			0.59±0.92		
rs12355840	TT	432	-0.77±1.02	-0.077 (-0.199, 0.045)	0.210	0.63±1.00	-0.078 (-0.211, 0.055)	0.250
	TC	81	-0.92±0.99			0.49±0.97		
	CC	4	-0.85±0.58			0.51±0.64		
	TC+CC	85	-0.91±0.97			0.49±0.95		
rs12402181	GG	325	-0.86±0.98	0.013 (-0.058, 0.084)	0.709	0.54±0.96	0.014 (-0.060, 0.088)	0.712
	GA	276	-0.95±1.00			0.46±0.97		
	AA	62	-0.86±0.99			0.56±0.95		
	GA+AA	338	-0.94±0.99			0.47±0.97		
	GG+GA	601	-0.91±0.99			0.50±0.97		
rs12451747	CC	140	-0.82±1.08	0.008 (-0.063, 0.079)	0.821	0.58±1.06	0.005 (-0.071, 0.081)	0.900
	CA	262	-0.88±0.97			0.52±0.94		
	AA	106	-0.61±1.02			0.78±1.01		
	CA+AA	368	-0.80±0.99			0.59±0.97		
	CC+CA	402	-0.86±1.01			0.54±0.98		
rs12803915	GG	469	-0.88±1.04	-0.026 (-0.118, 0.066)	0.581	0.53±1.02	-0.027 (-0.125, 0.071)	0.594
	GA	183	-0.89±0.86			0.51±0.85		
	AA	10	-1.52±0.77			-0.03±0.69		
	GA+AA	193	-0.92±0.87			0.48±0.85		
rs13299349	GG	532	-0.95±0.98	0.092 (-0.008, 0.192)	0.073	0.47±0.95	0.103 (-0.005, 0.211)	0.064
	GA	119	-0.64±1.03			0.76±1.00		
	AA	10	-0.98±0.78			0.49±0.81		
	GA+AA	129	-0.67±1.01			0.74±0.99		
rs1365477	CC	460	-0.77±1.00	-0.185	**0.019**	0.63±0.98	-0.227	**0.009**

	CT	49	**-1.05±1.10**[*]	(-0.340, -0.030)		**0.31±1.09**[*]	(-0.398, -0.056)	
	TT	2	0.36			1.65		
	CT+TT	51	**-1.02±1.11**[*]			**0.34±1.10**[*]		
rs1427865	GG	183	-0.70±1.01			0.70±0.98		
	GC	250	-0.88±1.01	-0.010 (-0.079, 0.059)	0.786	0.50±1.00	-0.007 (-0.083, 0.069)	0.860
	CC	84	-0.73±1.04			0.70±1.00		
	GC+CC	334	-0.84±1.02			0.55±1.00		
	GG+GC	433	-0.81±1.01			0.58±1.00		
rs2043556	TT	385	-0.93±0.98			0.47±0.95		
	TC	238	-0.84±1.03	-0.021 (-0.095, 0.053)	0.587	0.58±1.00	0.011 (-0.071, 0.093)	0.785
	CC	38	-0.95±0.87			0.52±0.89		
	TC+CC	276	-0.86±1.01			0.57±0.98		
rs2070960	CC	429	-0.91±0.99			0.49±0.97		
	CT	206	-0.89±1.01	0.068 (-0.010, 0.146)	0.089	0.53±0.98	0.081 (-0.003, 0.165)	0.064
	TT	29	-0.75±0.90			0.68±0.86		
	CT+TT	235	-0.87±1.00			0.55±0.97		
rs2114358	AA	307	-0.87±1.00			0.55±0.97		
	AG	291	-0.88±0.98	0.019 (-0.052, 0.090)	0.601	0.52±0.97	-0.004 (-0.080, 0.072)	0.925
	GG	61	-1.06±0.95			0.34±0.93		
	AG+GG	352	-0.91±0.98			0.49±0.96		
	AA+AG	598	-0.88±0.99			0.54±0.97		
rs2168518	GG	445	-0.89±1.01			0.52±0.98		
	GA	191	-0.89±0.96	0.018 (-0.062, 0.098)	0.666	0.51±0.94	4.95×10^{-4} (-0.086, 0.087)	0.991
	AA	29	-0.94±0.94			0.46±0.95		
	GA+AA	220	-0.89±0.96			0.50±0.94		
rs218662	AA	280	-0.87±1.02			0.52±1.00		
	AG	198	-0.68±1.00	0.020 (-0.054, 0.094)	0.603	0.72±0.97	0.025 (-0.057, 0.107)	0.553
	GG	41	-0.86±0.99			0.51±1.03		
	AG+GG	239	-0.71±1.00			0.69±0.98		
	AA+AG	478	-0.79±1.02			0.60±0.99		
rs2292832	TT	208	-0.77±1.00			0.62±0.99		
	TC	242	-0.86±1.01	-0.030 (-0.103, 0.043)	0.418	0.55±1.00	-0.030 (-0.110, 0.050)	0.467
	CC	59	-0.64±1.03			0.74±1.00		
	TC+CC	301	-0.81±1.02			0.59±1.00		
	TT+TC	450	-0.82±1.01			0.58±0.99		
rs231027	TT	156	-0.83±0.99			0.56±0.99		
	TC	256	-0.75±1.07	0.042 (-0.027, 0.111)	0.235	0.64±1.04	0.057 (-0.019, 0.133)	0.146
	CC	101	-0.90±0.89			0.53±0.88		
	TC+CC	357	-0.79±1.03			0.61±0.99		
	TT+TC	412	-0.78±1.04			0.61±1.02		
rs2594716	CC	279	-0.80±1.00			0.58±0.98		
	CT	195	-0.74±1.02	-0.084 (-0.158, -0.010)	**0.029**	0.66±1.01	-0.070 (-0.152, 0.012)	0.099
	TT	41	**-0.99±1.09**[*]			0.42±1.07		
	CT+TT	236	-0.78±1.03			0.62±1.02		
	CT+CC	474	**-0.78±1.01**[#]			**0.62±0.99**[#]		
rs2620381	AA	563	-0.91±1.00			0.50±0.97		
	AC	85	-0.91±0.90	0.068 (-0.055, 0.191)	0.276	0.50±0.89	0.076 (-0.055, 0.207)	0.260
	CC	5	-0.92±0.64			0.53±0.64		

	AC+CC	90	-0.91±0.89			0.50±0.87		
rs266437	CC	366	-0.82±1.01			0.58±0.99		
	CG	133	-0.73±1.02	0.019 (-0.071, 0.109)	0.683	0.66±0.99	0.005 (-0.095, 0.105)	0.922
	GG	16	-0.67±1.14			0.69±1.12		
	CG+GG	149	-0.72±1.03			0.66±1.00		
rs2910164	CC	245	-0.99±0.95			0.42±0.92		
	CG	321	-0.88±1.03	0.062 (-0.007, 0.131)	0.075	0.52±1.01	0.061 (-0.013, 0.135)	0.107
	GG	87	-0.67±0.94			0.74±0.91		
	CG+GG	408	-0.83±1.01			0.57±0.99		
	CC+CG	566	-0.93±1.00			0.48±0.97		
rs2967615	CC	258	-0.85±1.00			0.56±0.98		
	CT	213	-0.78±1.03	0.037 (-0.037, 0.111)	0.336	0.60±1.00	0.016 (-0.066, 0.098)	0.705
	TT	43	-0.58±1.04			0.79±1.07		
	CT+TT	256	-0.75±1.03			0.63±1.01		
	CC+CT	471	-0.82±1.01			0.58±0.99		
rs303064	CC	247	-0.77±1.03			0.63±1.00		
	CT	217	-0.85±0.99	0.038 (-0.035, 0.111)	0.306	0.54±0.98	0.035 (-0.045, 0.115)	0.387
	TT	49	-0.77±1.07			0.64±1.06		
	CT+TT	266	-0.83±1.01			0.56±0.99		
	CC+CT	464	-0.80±1.01			0.59±0.99		
rs34115976	CC	592	-0.91±1.00			0.50±0.97		
	CG	71	-0.80±0.91	-0.019 (-0.154, 0.116)	0.782	0.59±0.91	-0.030 (-0.177, 0.117)	0.688
	GG	3	-0.23±1.14			1.13±0.93		
	CG+GG	74	-0.78±0.92			0.61±0.91		
rs35770269	AA	324	-0.91±1.04			0.50±1.01		
	AT	272	-0.87±0.95	0.009 (-0.060, 0.078)	0.806	0.54±0.93	0.006 (-0.068, 0.080)	0.882
	TT	65	-0.95±0.94			0.46±0.89		
	AT+TT	337	-0.89±0.94			0.52±0.92		
	AA+AT	596	-0.89±1.00			0.52±0.98		
rs3823658	GG	503	-0.88±0.99			0.52±0.97		
	GA	142	-0.98±0.94	0.017 (-0.077, 0.111)	0.731	0.46±0.92	0.039 (-0.063, 0.141)	0.451
	AA	15	-0.59±1.15			0.82±1.11		
	GA+AA	157	-0.94±0.97			0.49±0.94		
rs4294864	CC	310	-0.81±1.02			0.57±1.00		
	CG	184	-0.76±1.03	0.024 (-0.058, 0.106)	0.578	0.66±1.01	0.052 (-0.040, 0.144)	0.270
	GG	23	-0.94±0.78			0.46±0.76		
	CG+GG	207	-0.78±1.01			0.64±0.99		
rs4919510	GG	200	-0.98±0.98			0.45±0.96		
	GC	329	-0.84±1.01	0.044 (-0.021, 0.109)	0.176	0.56±0.99	0.031 (-0.038, 0.100)	0.380
	CC	133	-0.88±0.94			0.52±0.92		
	GC+CC	462	-0.85±0.99			0.55±0.97		
	GG+GC	529	-0.90±1.00			0.52±0.98		
rs55680837	GG	163	-0.78±0.97			0.63±0.96		
	GC	252	-0.79±1.06	0.006 (-0.063, 0.075)	0.852	0.60±1.03	-8.81×10^{-5} (-0.075, 0.074)	0.998
	CC	98	-0.87±0.98			0.54±0.98		
	GC+CC	350	-0.81±1.03			0.58±1.01		
	GG+GC	415	-0.78±1.02			0.61±1.00		
rs56103835	CC	225	-0.74±0.99	-0.029	0.416	0.65±0.96	-0.029	0.464

	CT	223	-0.81±1.04	(-0.100, 0.042)		0.59±1.03	(-0.105, 0.047)	
	TT	68	-0.82±1.02			0.57±1.01		
	CT+TT	291	-0.82±1.04			0.58±1.02		
	CC+CT	448	-0.78±1.02			0.62±1.00		
rs56863230	GG	464	-0.79±1.01	-0.036 (-0.187, 0.115)	0.645	0.60±0.99	-0.022 (-0.189, 0.145)	0.799
	GC	48	-0.81±1.05			0.59±1.01		
	CC	2	-0.26±1.98			1.20±1.71		
	GC+CC	50	-0.79±1.07			0.61±1.03		
rs60432575	GG	580	-0.87±1.00	-0.099 (-0.221, 0.023)	0.114	0.54±0.97	-0.119 (-0.250, 0.012)	0.077
	GA	82	-1.04±0.89			0.37±0.90		
	AA	5	-1.19±1.39			0.14±1.33		
	GA+AA	87	-1.05±0.91			0.35±0.92		
rs60871950	GG	397	-0.90±1.00	0.011 (-0.065, 0.087)	0.772	0.52±0.98	-0.013 (-0.095, 0.069)	0.759
	GA	220	-0.89±0.99			0.50±0.95		
	AA	36	-0.92±0.98			0.47±0.99		
	GA+AA	256	-0.89±0.98			0.50±0.96		
rs61992671	AA	598	-0.88±1.00	-0.004 (-0.141, 0.133)	0.952	0.52±0.98	0.026 (-0.123, 0.175)	0.732
	AG	65	-1.04±0.92			0.40±0.88		
	GG	3	-0.70±0.51			0.75±0.39		
	AG+GG	68	-1.03±0.9			0.42±0.87		
rs6513497	TT	539	-0.90±0.98	0.012 (-0.098, 0.122)	0.831	0.51±0.96	0.028 (-0.090, 0.146)	0.636
	TG	97	-0.85±1.04			0.58±1.03		
	GG	9	-0.95±1.12			0.47±1.03		
	TG+GG	106	-0.86±1.04			0.57±1.02		
rs67106263	GG	505	-0.86±0.99	-0.055 (-0.153, 0.043)	0.266	0.55±0.96	-0.074 (-0.180, 0.032)	0.166
	GA	148	-0.99±0.98			0.40±0.97		
	AA	9	-0.88±1.09			0.54±1.04		
	GA+AA	157	-0.99±0.98			0.41±0.97		
rs7210937	GG	230	-0.92±1.03	0.031 (-0.034,0.096)	0.352	0.50±1.01	0.029 (-0.042,0.100)	0.423
	GC	324	-0.96±0.96			0.45±0.94		
	CC	112	-0.67±0.96			0.73±0.93		
	GC+ CC	436	-0.89±0.97			0.52±0.94		
	GG+ GC	554	-0.94±0.99			0.47±0.97		
rs725980	GG	286	-0.87±0.99	0.085 (0.007, 0.163)	**0.034**	0.53±0.97	0.092 (0.006, 0.178)	**0.038**
	GC	204	**-0.70±1.05***			**0.70±1.03***		
	CC	28	-0.79±0.95			0.62±0.95		
	GC+CC	232	**-0.71±1.04***			**0.69±1.02***		
rs73239138	GG	296	-0.82±0.98	-0.019 (-0.088, 0.050)	0.593	0.58±0.96	-0.005 (-0.079, 0.069)	0.891
	GA	291	-0.97±0.99			0.45±0.97		
	AA	66	-0.78±1.03			0.65±1.00		
	GA+AA	357	-0.93±1.00			0.49±0.97		
	GG+GA	587	-0.89±0.99			0.52±0.96		
rs73410309	GG	575	-0.91±0.99	0.095 (-0.032, 0.222)	0.141	0.50±0.97	0.094 (-0.043, 0.231)	0.178
	GC	89	-0.82±0.97			0.59±0.94		
	CC	2	-1.62			-0.07		
	GC+CC	91	-0.83±0.97			0.58±0.94		
rs75330474	CC	593	-0.88±0.98	-0.042 (-0.183, 0.099)	0.558	0.54±0.95	-0.056 (-0.209, 0.097)	0.474
	CT	67	-1.02±1.04			0.39±1.04		

	TT	2	-1.17±1.10			0.16±1.25		
	CT+TT	69	-1.02±1.03			0.38±1.04		
rs77639117	AA	478	-0.79±1.03	-0.074 (-0.246, 0.098)	0.404	0.60±1.01	-0.058 (-0.248, 0.132)	0.553
	AT	39	-0.85±0.87			0.56±0.84		
	TT	1	-1.46			0.07		
	AT+TT	40	-0.86±0.86			0.55±0.84		
rs78861479	GG	462	-0.79±1.02	-0.494 (-1.061, 0.072)	0.087	0.60±1.01	-0.480 (-1.095, 0.135)	0.126
	GA	10	-0.26±1.01			1.12±0.90		
rs79402775	GG	477	-0.89±1.01	-0.028 (-0.116, 0.060)	0.538	0.51±0.99	-0.003 (-0.099, 0.093)	0.957
	GA	165	-0.90±0.94			0.53±0.90		
	AA	15	-0.87±0.88			0.60±0.87		
	GA+AA	180	-0.89±0.93			0.54±0.90		
rs7954025	AA	286	-0.83±1.00	0.040 (-0.034, 0.114)	0.299	0.58±0.99	0.029 (-0.053, 0.111)	0.488
	AG	188	-0.75±1.03			0.63±1.01		
	GG	40	-0.81±1.04			0.59±1.00		
	AG+GG	228	-0.76±1.03			0.62±1.00		
	AA+AG	474	-0.80±1.01			0.60±0.99		
rs8016027	CC	319	-0.81±1.05	0.043 (-0.037, 0.123)	0.301	0.59±1.04	0.033 (-0.057, 0.123)	0.467
	CT	170	-0.75±0.97			0.62±0.93		
	TT	27	-0.88±0.95			0.53±0.93		
	CT+TT	197	-0.77±0.96			0.61±0.93		
rs878718	AA	154	-0.82±1.08	0.023 (-0.048, 0.094)	0.517	0.55±1.07	0.047 (-0.029, 0.123)	0.239
	AG	269	-0.78±1.01			**0.63±0.98**[*]		
	GG	92	-0.82±0.94			0.59±0.93		
	AG+GG	361	-0.79±0.99			**0.62±0.96**[*]		
	AG+AA	423	-0.79±1.03			0.60±1.00		

[a] Ln-transformed.
[*] *P* <0.05 when compared with wild-type homozygotes by multivariate analysis of covariance with adjustment for age, gender, smoking status, pack-years of smoking, drinking status, factory location, and BMI.
[#] *P* <0.05 when compared with mutant homozygotes by multivariate analysis of covariance with adjustment for age, gender, smoking status, pack-years of smoking, drinking status, factory location, and BMI.
[b] Z-Score standardization after ln-transformed.
[c] Multivariate linear regression with adjustment for age, gender, smoking status, pack-years of smoking, drinking status, factory location, and BMI.

Table S7 Modifying effects of SNPs on the associations [β_{std} (95% CI)] of benzene and BTX mixture with OTM and Tail DNA%.

SNP	Genotype	Associations of benzene CE levels [a] with OTM [a]		Associations of benzene CE levels [a] with Tail DNA% [a]		Associations of wqs index [a] with OTM		Associations of wqs index [a] with Tail DNA%	
		β_{std} (95%CI)	$P_{interaction}$ [b]	β_{std} (95%CI)	$P_{interaction}$ [b]	β_{std} (95%CI)	$P_{interaction}$ [b]	β_{std} (95%CI)	$P_{interaction}$ [b]
rs11191980	TT	0.110 (0.043, 0.177)*	0.688	0.135 (0.061, 0.209)**	0.902	0.123 (0.053, 0.193)*	0.623	0.151 (0.070, 0.232)**	0.960
	TC+CC	0.078 (-0.067, 0.223)		0.123 (-0.026, 0.272)		0.081 (-0.069, 0.230)		0.132 (-0.027, 0.290)	
rs112250173	TT	0.074 (0.005, 0.143)*	0.354	0.102 (0.029, 0.175)*	0.495	0.090 (0.017, 0.162)*	0.406	0.122 (0.043, 0.201)*	0.691
	TG+GG	0.186 (0.049, 0.323)*		0.233 (0.066, 0.400)*		0.188 (0.044, 0.332)*		0.236 (0.059, 0.414)*	
rs11237828	TT	0.102 (-0.010, 0.214)	0.142	0.141 (0.019, 0.263)*	0.120	0.114 (-0.003, 0.231)	0.695	0.155 (0.023, 0.286)*	0.571
	TC	0.001 (-0.089, 0.091)		0.015 (-0.081, 0.111)		-0.006 (-0.101, 0.090)		0.006 (-0.099, 0.112)	
	CC	0.154 (0.052, 0.256)*		0.194 (0.084, 0.304)*		0.162 (0.059, 0.264)*		0.205 (0.092, 0.318)*	
rs11614913	TT	-0.017 (-0.139, 0.105)	0.113	0.041 (-0.084, 0.166)	0.395	-0.015 (-0.141, 0.111)	**0.043**	0.047 (-0.088, 0.181)	0.235
	TC	0.095 (0.017, 0.173)*		0.115 (0.031, 0.199)*		0.094 (0.011, 0.177)*		0.114 (0.022, 0.206)*	
	CC	0.090 (-0.028, 0.208)		0.099 (-0.040, 0.238)		0.119 (-0.001, 0.239)		0.126 (-0.020, 0.273)	
rs12220909	GG	0.055 (-0.021, 0.131)	0.432	0.082 (-0.002, 0.166)	0.493	0.060 (-0.022, 0.141)	0.489	0.087 (-0.004, 0.179)	0.609
	GC+CC	0.008 (-0.004, 0.164)		0.111 (0.023, 0.199)*		0.091 (0.002, 0.180)*		0.124 (0.029, 0.218)*	
rs12355840	TT	0.105 (0.038, 0.172)*	0.321	0.138 (0.064, 0.212)**	0.367	0.116 (0.045, 0.187)*	0.428	0.153 (0.072, 0.234)**	0.519
	TC+CC	0.016 (-0.131, 0.163)		0.027 (-0.128, 0.182)		0.032 (-0.119, 0.182)		0.044 (-0.118, 0.206)	
rs12402181	GG	0.086 (0.010, 0.162)*	0.914	0.115 (0.033, 0.197)*	0.937	0.091 (0.012, 0.170)*	0.837	0.123 (0.034, 0.212)*	0.789
	GA	0.001 (-0.103, 0.105)		0.022 (-0.090, 0.134)		0.010 (-0.102, 0.122)		0.028 (-0.094, 0.150)	
	AA	0.110 (-0.092, 0.312)		0.132 (-0.080, 0.344)		0.109 (-0.081, 0.300)		0.134 (-0.068, 0.336)	
rs12451747	CC	0.203 (0.087, 0.319)*	0.052	0.210 (0.087, 0.333)*	0.119	0.213 (0.095, 0.332)**	**0.038**	0.224 (0.093, 0.354)*	0.112
	CA	0.055 (-0.025, 0.135)		0.095 (0.005, 0.185)*		0.073 (-0.013, 0.160)		0.118 (0.020, 0.217)*	
	AA	0.060 (-0.099, 0.219)		0.091 (-0.078, 0.260)		0.066 (-0.094, 0.226)		0.105 (-0.072, 0.283)	
rs12803915	GG	0.080 (0.009, 0.151)*	**0.030**	0.109 (0.035, 0.183)*	**0.022**	0.081 (0.007, 0.155)*	**0.048**	0.110 (0.029, 0.191)*	0.050
	GA+AA	0.034 (-0.062, 0.130)		0.052 (-0.054, 0.158)		0.042 (-0.060, 0.144)		0.062 (-0.054, 0.178)	
rs13299349	GG	0.069 (0.006, 0.132)*	0.696	0.100 (0.031, 0.169)*	0.854	0.072 (0.005, 0.139)*	0.786	0.104 (0.030, 0.179)*	0.991

	GA+AA	0.037 (-0.100, 0.174)		0.057 (-0.084, 0.198)		0.045 (-0.097, 0.186)		0.065 (-0.085, 0.215)	
rs1365477	CC	0.115 (0.052, 0.178)**	0.261	0.139 (0.070, 0.208)**	0.361	0.128 (0.061, 0.195)**	0.223	0.156 (0.080, 0.232)**	0.316
	CT+TT	-0.093 (-0.311, 0.125)		-0.024 (-0.269, 0.221)		-0.095 (-0.306, 0.116)		-0.014 (-0.259, 0.231)	
rs1427865	GG	0.083 (-0.013, 0.179)	0.637	0.113 (0.015, 0.211)*	0.721	0.104 (0.004, 0.203)*	0.729	0.140 (0.035, 0.245)*	0.708
	GC	0.126 (0.032, 0.220)*		0.165 (0.059, 0.271)*		0.130 (0.031, 0.229)*		0.174 (0.058, 0.290)*	
	CC	-0.051 (-0.206, 0.104)		-0.073 (-0.247, 0.101)		-0.043 (-0.199, 0.113)		-0.075 (-0.255, 0.104)	
rs2043556	TT	0.028 (-0.052, 0.108)	0.255	0.057 (-0.029, 0.143)	0.274	0.037 (-0.048, 0.123)	0.357	0.067 (-0.028, 0.161)	0.401
	TC+CC	0.125 (0.045, 0.206)*		0.151 (0.063, 0.238)*		0.126 (0.041, 0.211)*		0.151 (0.056, 0.246)*	
rs2070960	CC	0.047 (-0.024, 0.118)	0.965	0.082 (0.006, 0.158)*	0.668	0.019 (-0.045, 0.082)	0.920	0.090 (0.008, 0.172)*	0.572
	CT+TT	0.097 (-0.001, 0.196)		0.114 (0.008, 0.221)*		0.054 (-0.020, 0.128)		0.115 (-0.002, 0.232)	
rs2114358	AA	0.052 (-0.034, 0.138)	0.276	0.069 (-0.023, 0.161)	0.315	0.051 (-0.040, 0.142)	0.712	0.067 (-0.031, 0.165)	0.518
	AG	0.052 (-0.026, 0.130)		0.089 (0.001, 0.177)*		0.061 (-0.022, 0.144)		0.101 (0.006, 0.196)*	
	GG	0.259 (-0.023, 0.541)		0.286 (-0.002, 0.574)		0.279 (-0.002, 0.561)		0.320 (0.025, 0.615)*	
rs2168518	GG	0.042 (-0.025, 0.109)	0.441	0.063 (-0.006, 0.132)	0.511	0.050 (-0.020, 0.120)	0.517	0.073 (-0.002, 0.149)	0.581
	GA+AA	0.117 (0.005, 0.229)*		0.163 (0.036, 0.290)*		0.106 (-0.011, 0.224)		0.148 (0.011, 0.286)*	
rs218662	AA	0.108 (0.020, 0.196)*	0.714	0.128 (0.030, 0.226)*	0.551	0.124 (0.030, 0.217)*	0.987	0.143 (0.036, 0.250)*	0.428
	AG	0.049 (-0.047, 0.145)		0.076 (-0.026, 0.178)		0.052 (-0.049, 0.153)		0.089 (-0.022, 0.199)	
	GG	0.138 (-0.056, 0.332)		0.230 (0.032, 0.428)*		0.158 (-0.023, 0.339)		0.261 (0.043, 0.478)*	
rs2292832	TT	0.095 (-0.011, 0.201)	0.870	0.124 (0.008, 0.240)*	0.931	0.105 (-0.005, 0.215)	0.848	0.138 (0.015, 0.262)*	0.978
	TC	0.121 (0.039, 0.203)*		0.153 (0.061, 0.245)*		0.130 (0.042, 0.219)*		0.166 (0.065, 0.267)*	
	CC	-0.011 (-0.209, 0.187)		0.019 (-0.185, 0.223)		0.025 (-0.166, 0.216)		0.070 (-0.132, 0.273)	
rs231027	TT	0.115 (-0.001, 0.231)	0.922	0.166 (0.033, 0.299)*	0.927	0.121 (0.000, 0.243)	0.912	0.182 (0.038, 0.327)*	0.660
	TC	0.065 (-0.023, 0.153)		0.078 (-0.016, 0.172)		0.075 (-0.017, 0.167)		0.090 (-0.011, 0.192)	
	CC	0.151 (0.026, 0.276)*		0.198 (0.067, 0.329)*		0.169 (0.040, 0.298)*		0.224 (0.084, 0.364)*	
rs2594716	CC	0.116 (0.040, 0.192)*	0.926	0.147 (0.063, 0.231)*	0.985	0.121 (0.041, 0.202)*	0.881	0.156 (0.064, 0.248)*	0.638
	CT	0.055 (-0.045, 0.155)		0.075 (-0.035, 0.185)		0.082 (-0.027, 0.190)		0.108 (-0.013, 0.228)	
	TT	0.104 (-0.372, 0.580)		0.286 (-0.233, 0.805)		0.161 (-0.244, 0.566)		0.334 (-0.112, 0.779)	

rs2620381	AA	0.055 (-0.008, 0.118)	0.673	0.080 (0.011, 0.149)*	0.538	0.058 (-0.010, 0.125)	0.656	0.083 (0.008, 0.157)*	0.514
	AC+CC	0.068 (-0.085, 0.221)		0.118 (-0.049, 0.285)		0.077 (-0.076, 0.229)		0.128 (-0.042, 0.298)	
rs266437	CC	0.136 (0.069, 0.203)**	**0.019**	0.159 (0.086, 0.232)**	**0.024**	0.150 (0.079, 0.221)**	**0.017**	0.177 (0.098, 0.257)**	**0.021**
	CG+GG	-0.030 (-0.167, 0.107)		0.023 (-0.132, 0.178)		-0.020 (-0.163, 0.123)		0.032 (-0.133 ,0.197)	
rs2910164	CC	0.075 (-0.015, 0.165)	0.768	0.086 (-0.010, 0.182)	0.553	0.097 (0.004, 0.190)*	0.844	0.105 (0.004, 0.207)*	0.808
	CG	0.042 (-0.054, 0.138)		0.094 (-0.008, 0.196)		0.036 (-0.065, 0.137)		0.092 (-0.019, 0.203)	
	GG	0.087 (-0.038, 0.212)		0.094 (-0.053, 0.241)		0.084 (-0.043, 0.212)		0.092 (-0.062, 0.247)	
rs2967615	CC	0.111 (0.019, 0.203)*	0.918	0.109 (0.007, 0.211)*	0.781	0.129 (0.028, 0.229)*	0.634	0.130 (0.016, 0.243)*	0.872
	CT	0.081 (-0.007, 0.169)		0.135 (0.039, 0.231)*		0.088 (-0.002, 0.178)		0.147 (0.046, 0.248)*	
	TT	0.145 (-0.100, 0.390)		0.203 (-0.075, 0.481)		0.159 (-0.076, 0.395)		0.220 (-0.054, 0.495)	
rs303064	CC	0.065 (-0.017, 0.147)	0.532	0.068 (-0.020, 0.156)	0.419	0.087 (0.000, 0.174)	0.477	0.095 (-0.002, 0.191)	0.440
	CT	0.148 (0.048, 0.248)*		0.223 (0.109, 0.337)**		0.145 (0.039, 0.251)*		0.228 (0.104, 0.352)**	
	TT	0.098 (-0.123, 0.319)		0.089 (-0.136, 0.314)		0.120 (-0.096, 0.337)		0.107 (-0.121, 0.335)	
rs34115976	CC	0.072 (0.009, 0.135)*	0.331	0.095 (0.028, 0.162)*	0.616	0.074 (0.009, 0.140)*	0.476	0.098 (0.025, 0.170)*	0.845
	CG+GG	0.040 (-0.109, 0.189)		0.082 (-0.077, 0.241)		0.062 (-0.087, 0.211)		0.112 (-0.052, 0.276)	
rs35770269	AA	0.075 (-0.005, 0.155)	0.266	0.092 (0.008, 0.176)*	0.327	0.084 (0.000, 0.168)	0.157	0.102 (0.011, 0.193)*	0.149
	AT	0.047 (-0.041, 0.135)		0.094 (-0.004, 0.192)		0.040 (-0.052, 0.133)		0.091 (-0.016, 0.198)	
	TT	0.022 (-0.223, 0.267)		0.003 (-0.244, 0.250)		0.046 (-0.185, 0.278)		0.017 (-0.222, 0.255)	
rs3823658	GG	0.084 (0.019, 0.149)*	0.695	0.115 (0.044, 0.186)*	0.732	0.090 (0.021, 0.159)*	0.694	0.125 (0.048, 0.202)*	0.668
	GA+AA	4.43×10^{-4} (-0.121, 0.122)		0.005 (-0.124, 0.134)		0.002 (-0.121, 0.125)		0.004 (-0.130, 0.138)	
rs4294864	CC	0.089 (0.006, 0.173)*	0.826	0.117 (0.024, 0.210)*	0.649	0.100 (0.011, 0.190)*	0.865	0.129 (0.027, 0.232)*	0.681
	CG+GG	0.125 (0.033, 0.217)*		0.156 (0.058, 0.254)*		0.137 (0.040, 0.235)*		0.174 (0.067, 0.282)*	
rs4919510	GG	0.054 (-0.060, 0.168)	0.829	0.081 (-0.041, 0.203)	0.822	0.057 (-0.063, 0.177)	0.552	0.084 (-0.047, 0.215)	0.651
	GC	0.027 (-0.051, 0.105)		0.057 (-0.027, 0.141)		0.029 (-0.053, 0.111)		0.060 (-0.030, 0.150)	
	CC	0.110 (-0.013, 0.233)		0.122 (-0.015, 0.259)		0.115 (-0.013, 0.243)		0.126 (-0.019, 0.272)	
rs55680837	GG	0.078 (-0.006, 0.162)	0.604	0.080 (-0.010, 0.170)	0.540	0.074 (-0.014, 0.162)	0.191	0.078 (-0.020, 0.177)	0.153
	GC	0.063 (-0.035, 0.161)		0.105 (-0.001, 0.211)		0.076 (-0.026, 0.178)		0.120 (0.006, 0.233)*	

	CC	0.232 (0.073, 0.391)*		0.286 (0.106, 0.466)*		0.274 (0.111, 0.438)*		0.349 (0.155, 0.544)*	
rs56103835	CC	0.069 (-0.011, 0.149)	0.334	0.077 (-0.007, 0.161)	0.200	0.078 (-0.006, 0.162)	0.765	0.087 (-0.005, 0.179)	0.455
	CT	0.132 (0.036, 0.228)		0.173 (0.065, 0.281)*		0.145 (0.046, 0.245)*		0.187 (0.070, 0.304)*	
	TT	0.126 (-0.160, 0.412)		0.222 (-0.101, 0.545)		0.000 (-0.009, 0.009)		0.260 (-0.061, 0.580)	
rs56863230	GG	0.098 (0.033, 0.163)*	0.732	0.127 (0.054, 0.200)*	0.889	0.112 (0.043, 0.181)*	0.545	0.146 (0.067, 0.225)**	0.683
	GC+CC	0.036 (-0.180, 0.252)		0.079 (-0.137, 0.295)		0.027 (-0.181, 0.235)		0.075 (-0.140, 0.289)	
rs60432575	GG	0.071 (0.008, 0.134)*	0.585	0.094 (0.027, 0.161)*	0.657	0.077 (0.012, 0.143)*	0.601	0.101 (0.030, 0.173)*	0.697
	GA+AA	0.030 (-0.121, 0.181)		0.092 (-0.088, 0.272)		0.019 (-0.132, 0.171)		0.077 (-0.111, 0.264)	
rs60871950	GG	0.092 (0.014, 0.170)*	0.399	0.111 (0.027, 0.195)*	0.343	0.095 (0.012, 0.178)*	0.419	0.116 (0.024, 0.207)*	0.363
	GA+AA	0.035 (-0.049, 0.120)		0.065 (-0.026, 0.157)		0.041 (-0.048, 0.130)		0.074 (-0.025, 0.172)	
rs61992671	AA	0.068 (0.007, 0.129)*	0.769	0.091 (0.026, 0.156)*	0.880	0.074 (0.010, 0.138)*	0.526	0.098 (0.028, 0.169)*	0.611
	AG+GG	0.063 (-0.113, 0.239)		0.143 (-0.051, 0.337)		0.056 (-0.123, 0.234)		0.142 (-0.062, 0.346)	
rs6513497	TT	0.049 (-0.014, 0.112)	0.398	0.081 (0.012, 0.150)*	0.402	0.053 (-0.014, 0.120)	0.436	0.087 (0.013, 0.160)*	0.474
	TG+GG	0.137 (-0.022, 0.296)		0.152 (-0.017, 0.321)		0.148 (-0.018, 0.314)		0.162 (-0.019, 0.342)	
rs67106263	GG	0.056 (-0.011, 0.123)	0.142	0.081 (0.010, 0.152)*	0.158	0.061 (-0.010, 0.133)	0.179	0.089 (0.012, 0.166)*	0.206
	GA+AA	0.113 (0.007, 0.219)*		0.150 (0.025, 0.275)*		0.117 (0.010, 0.224)*		0.151 (0.022, 0.281)*	
rs7210937	GG	0.069 (-0.044, 0.183)	0.451	0.081 (-0.041, 0.202)	0.511	0.078 (-0.039, 0.194)	0.481	0.085 (-0.043, 0.213)	0.613
	GC	0.062 (-0.060, 0.184)		0.115 (-0.023, 0.252)		0.068 (-0.056, 0.192)		0.129 (-0.015, 0.273)	
	CC	0.072 (-0.009, 0.153)		0.102 (0.015, 0.189)*		0.073 (-0.012, 0.157)		0.104 (0.012, 0.197)*	
rs725980	GG	0.113 (0.030, 0.197)*	0.828	0.147 (0.054, 0.241)*	0.629	0.126 (0.035, 0.216)*	0.992	0.163 (0.059, 0.267)*	0.818
	GC+CC	0.081 (-0.005, 0.167)		0.107 (0.015, 0.199)*		0.090 (-0.001, 0.181)		0.122 (0.023, 0.220)*	
rs73239138	GG	0.070 (-0.020, 0.160)	0.973	0.113 (0.013, 0.213)*	0.977	0.065 (-0.029, 0.160)	0.727	0.029 (-0.044, 0.101)	0.692
	GA	0.083 (-0.009, 0.175)		0.103 (0.005, 0.201)*		0.095 (-0.001, 0.191)		0.109 (0.002, 0.217)*	
	AA	0.092 (-0.049, 0.233)		0.103 (-0.042, 0.248)		0.114 (-0.029, 0.258)		0.114 (0.009, 0.219)*	
rs73410309	GG	0.070 (0.009, 0.131)*	0.452	0.097 (0.032, 0.162)*	0.500	0.073 (0.009, 0.136)*	0.537	0.101 (0.030, 0.172)*	0.624
	GC+CC	0.005 (-0.189, 0.199)		0.033 (-0.171, 0.237)		0.024 (-0.171, 0.219)		0.055 (-0.155, 0.265)	
rs75330474	CC	0.065 (0.008, 0.122)*	0.727	0.094 (0.033, 0.155)*	0.821	0.072 (0.013, 0.132)*	0.703	0.103 (0.037, 0.169)*	0.792

	CT+TT	0.054 (-0.258, 0.366)		0.046 (-0.281, 0.373)		0.061 (-0.247, 0.368)		0.053 (-0.278, 0.384)	
rs77639117	AA	0.096 (0.031, 0.161)*	0.830	0.127 (0.056, 0.198)**	0.817	0.107 (0.038, 0.176)*	0.842	0.143 (0.065, 0.221)**	0.857
	AT+TT	0.086 (-0.036, 0.208)		0.073 (-0.054, 0.200)		0.113 (0.002, 0.224)*		0.098 (-0.023, 0.219)	
rs78861479	GG	0.099 (0.032, 0.165)*	0.114	0.131 (0.057, 0.204)**	0.103	0.105 (0.034, 0.177)*	0.299	0.140 (0.060, 0.221)*	0.239
	GA	-0.131 (-0.591, 0.330)		-0.115 (-0.481, 0.252)		-0.138 (-0.168, -0.107)**		-0.127 (-0.152, -0.102)**	
rs79402775	GG	0.068 (-0.010, 0.146)	0.951	0.095 (0.011, 0.179)*	0.977	0.079 (-0.004, 0.163)	0.845	0.107 (0.015, 0.200)*	0.798
	GA+AA	0.068 (-0.008, 0.144)		0.097 (0.015, 0.179)*		0.063 (-0.015, 0.141)		0.094 (0.008, 0.181)*	
rs7954025	AA	0.113 (0.035, 0.191)*	0.489	0.149 (0.063, 0.235)*	0.648	0.129 (0.044, 0.214)*	0.917	0.172 (0.076, 0.268)**	0.905
	AG	0.081 (-0.029, 0.191)		0.096 (-0.027, 0.219)		0.098 (-0.015, 0.211)		0.117 (-0.013, 0.246)	
	GG	0.164 (-0.050, 0.378)		0.166 (-0.052, 0.384)		0.181 (-0.024, 0.385)		0.186 (-0.030, 0.402)	
rs8016027	CC	0.108 (0.030, 0.186)*	0.893	0.143 (0.059, 0.227)*	0.462	0.125 (0.041, 0.208)*	0.916	0.166 (0.072, 0.259)*	0.529
	CT+TT	0.066 (-0.025, 0.158)		0.082 (-0.022, 0.185)		0.069 (-0.028, 0.166)		0.086 (-0.026, 0.199)	
rs878718	AA	0.152 (0.034, 0.270)*	0.353	0.189 (0.056, 0.322)*	0.278	0.186 (0.065, 0.307)*	0.210	0.225 (0.085, 0.365)*	0.153
	AG	0.083 (0.003, 0.163)*		0.114 (0.028, 0.200)*		0.092 (0.007, 0.177)*		0.128 (0.034, 0.222)*	
	GG	0.077 (-0.068, 0.222)		0.103 (-0.068, 0.274)		0.074 (-0.077, 0.226)		0.107 (-0.077, 0.290)	

[a] Z-Score standardization after ln-transformed.

[b] $P_{interaction}$ was calculated by modeling an interaction term of benzene CE levels (continuous) and SNP genotypes (categorical) into generalized linear models with adjustment for age, gender, smoking status, pack-years of smoking, drinking status, factory location, and BMI.

Significant levels of the associations, which was calculated by generalized linear models with adjustment for age, gender, smoking status, pack-years of smoking, drinking status, factory location, and BMI. were annotated as ** $P_{trend} < 0.001$; and * $P_{trend} < 0.05$;

Table S8. Modifying effects of benzene exposure on the associations [β_{std} (95% CI)] of SNPs (continuous) with OTM and Tail DNA%.

SNPs	Benzene exposure levels [a]	Association of SNPs with OTM [a]		Association of SNPs with Tail DNA% [a]		Wqs index [a]	Association of SNPs with OTM [a]		Association of SNPs with Tail DNA% [a]	
		β_{std} (95%CI)	$P_{interaction}$ [c]	β_{std} (95%CI)	$P_{interaction}$ [c]		β_{std} (95%CI)	$P_{interaction}$ [c]	β_{std} (95%CI)	$P_{interaction}$ [c]
rs11191980	Low	-0.007 (-0.140, 0.125)	0.551	-0.051 (-0.198, 0.096)	0.947	Low	-0.090 (-0.221, 0.040)	0.334	-0.016 (-0.161, 0.130)	0.286
	High	-0.079 (-0.214, 0.055)		-0.049 (-0.194, 0.096)		High	0.006 (-0.129, 0.142)		-0.120 (-0.264, 0.024)	
rs112250173	Low	0.060 (-0.092, 0.212)	0.995	0.058 (-0.112, 0.227)	0.612	Low	0.083 (-0.048, 0.214)	0.779	0.020 (-0.152, 0.192)	0.880
	High	0.066 (-0.068, 0.199)		-0.003 (-0.148, 0.142)		High	0.050 (-0.106, 0.205)		0.034 (-0.106, 0.174)	
rs11237828	Low	0.018 (-0.078, 0.114)	0.904	0.008 (-0.096, 0.113)	0.865	Low	0.034 (-0.050, 0.119)	0.800	-0.006 (-0.114 ,0.102)	0.516
	High	0.019 (-0.066, 0.104)		0.032 (-0.059, 0.123)		High	0.007 (-0.090, 0.104)		0.047 (-0.039, 0.133)	
rs11614913	Low	-0.057 (-0.154, 0.039)	**0.039**	-0.051 (-0.155, 0.054)	0.069	Low	-0.058 (-0.156, 0.039)	0.126	-0.073 (-0.180, 0.034)	0.130
	High	0.022 (-0.067, 0.112)		-0.025 (-0.121, 0.071)		High	0.040 (-0.049, 0.129)		0.029 (-0.064, 0.121)	
rs12220909	Low	0.131 (0.012, 0.249)*	**0.024**	0.149 (0.021, 0.277)*	**0.020**	Low	0.138 (0.019, 0.256)*	**0.013**	0.123 (-0.011, 0.256)	0.069
	High	-0.043 (-0.140, 0.055)		-0.042 (-0.146, 0.063)		High	-0.055 (-0.152, 0.043)		-0.024 (-0.124, 0.075)	
rs12355840	Low	0.012 (-0.160, 0.184)	0.139	0.026 (-0.166, 0.218)	0.154	Low	0.025 (-0.142, 0.192)	0.079	0.029 (-0.153, 0.211)	0.065
	High	-0.166 (-0.328, -0.004)*		-0.172 (-0.347, 0.004)		High	-0.191 (-0.361, -0.020)*		-0.223 (-0.411, -0.036)*	
rs12402181	Low	0.024 (-0.077, 0.124)	0.882	0.014 (-0.095, 0.122)	0.844	Low	0.032 (-0.068, 0.132)	0.704	-0.006 (-0.097, 0.085)	0.669
	High	0.009 (-0.081, 0.099)		0.026 (-0.070, 0.123)		High	-0.006 (-0.097, 0.085)		0.032 (-0.068, 0.132)	
rs12451747	Low	0.082 (-0.024, 0.188)	0.077	0.067 (-0.050, 0.184)	0.180	Low	-0.092 (-0.197, 0.002)	**0.032**	-0.084 (-0.200, 0.031)	**0.034**
	High	-0.054 (-0.142, 0.034)		-0.043 (-0.136, 0.049)		High	0.062 (-0.028, 0.028)		0.066 (-0.025, 0.158)	
rs12803915	Low	0.050 (-0.084, 0.183)	0.107	0.058 (-0.087, 0.202)	0.094	Low	0.029 (-0.108, 0.165)	0.243	0.020 (-0.128, 0.168)	0.334
	High	-0.095 (-0.212, 0.021)		-0.107 (-0.232, 0.018)		High	-0.078 (-0.192, 0.035)		-0.075 (-0.195, 0.044)	
rs13299349	Low	0.143 (-0.019, 0.305)	0.259	0.129 (-0.046, 0.305)	0.488	Low	0.148 (-0.013, 0.310)	0.232	0.147 (-0.035, 0.329)	0.347
	High	0.033 (-0.085, 0.151)		0.059 (-0.067, 0.185)		High	0.031 (-0.088, 0.149)		0.053 (-0.068, 0.174)	
rs1365477	Low	-0.174 (-0.404, 0.055)	0.878	-0.235 (-0.490, 0.019)	0.673	Low	-0.139 (-0.368, 0.090)	0.707	-0.217 (-0.478, 0.044)	0.919
	High	-0.150 (-0.346, 0.047)		-0.163 (-0.377, 0.050)		High	-0.185 (-0.383, 0.013)		-0.205 (-0.410, 0.000)	
rs1427865	Low	-0.050 (-0.151, 0.052)	0.229	-0.03 (-0.144, 0.083)	0.480	Low	-0.049 (-0.151, 0.053)	0.252	-0.017 (-0.128, 0.095)	0.608

	High	0.041 (-0.046, 0.128)		0.031 (-0.064, 0.125)		High	0.031 (-0.057, 0.119)		0.018 (-0.076, 0.113)	
rs2043556	Low	-0.043 (-0.152, 0.066)	0.365	-0.018 (-0.135, 0.100)	0.318	Low	-0.050 (-0.159, 0.059)	0.298	-0.001 (-0.122, 0.120)	0.581
	High	0.034 (-0.062, 0.130)		0.076 (-0.026, 0.178)		High	0.040 (-0.055, 0.136)		0.053 (-0.045, 0.152)	
rs2070960	Low	0.077 (-0.039, 0.193)	0.813	0.107 (-0.018, 0.232)	0.498	Low	0.097 (-0.020, 0.214)	0.447	0.155 (0.026, 0.285)*	0.062
	High	0.054 (-0.046, 0.154)		0.043 (-0.064, 0.151)		High	0.037 (-0.062, 0.136)		-0.007 (-0.109, 0.094)	
rs2114358	Low	0.013 (-0.086, 0.112)	0.690	-0.013 (-0.119, 0.094)	0.630	Low	0.016 (-0.083, 0.116)	0.824	-0.024 (-0.133, 0.086)	0.420
	High	0.043 (-0.051, 0.137)		0.022 (-0.078, 0.123)		High	0.027 (-0.067, 0.121)		0.036 (-0.060, 0.132)	
rs2168518	Low	-0.014 (-0.126, 0.098)	0.276	-0.019 (-0.140, 0.103)	0.486	Low	0.000 (-0.112, 0.111)	0.469	-0.015 (-0.137, 0.108)	0.493
	High	0.062 (-0.047, 0.171)		0.026 (-0.091, 0.143)		High	0.043 (-0.068, 0.153)		0.036 (-0.079, 0.151)	
rs218662	Low	0.033 (-0.071, 0.137)	0.765	0.026 (-0.090, 0.142)	0.942	Low	0.037 (-0.068, 0.142)	0.700	0.018 (-0.099, 0.135)	0.983
	High	0.008 (-0.096, 0.112)		0.034 (-0.078, 0.147)		High	0.015 (-0.088, 0.119)		0.023 (-0.086, 0.132)	
rs2292832	Low	-0.055 (-0.163, 0.053)	0.326	-0.054 (-0.175, 0.066)	0.406	Low	-0.060 (-0.171, 0.051)	0.401	-0.071 (-0.192, 0.050)	0.274
	High	0.019 (-0.074, 0.112)		0.019 (-0.081, 0.120)		High	0.000 (-0.092, 0.091)		0.010 (-0.088, 0.108)	
rs231027	Low	0.026 (-0.072, 0.124)	0.611	0.025 (-0.084, 0.134)	0.353	Low	0.036 (-0.062, 0.135)	0.862	0.052 (-0.057, 0.161)	0.782
	High	0.058 (-0.033, 0.148)		0.090 (-0.009, 0.188)		High	0.045 (-0.045, 0.136)		0.065 (-0.032, 0.162)	
rs2594716	Low	-0.110 (-0.216, -0.004)*	0.289	-0.12 (-0.238, -0.001)*	0.123	Low	-0.101 (-0.208, 0.005)	0.422	-0.071 (-0.187, 0.045)	0.759
	High	-0.027 (-0.126, 0.072)		0.016 (-0.092, 0.123)		High	-0.034 (-0.134, 0.066)		-0.039 (-0.148, 0.071)	
rs2620381	Low	0.077 (-0.096, 0.249)	0.723	0.071 (-0.115, 0.257)	0.929	Low	0.049 (-0.126, 0.223)	0.807	0.016 (-0.177, 0.209)	0.276
	High	0.029 (-0.132, 0.190)		0.053 (-0.120, 0.225)		High	0.081 (-0.079, 0.241)		0.170 (0.007, 0.332)*	
rs266437	Low	0.115 (-0.014, 0.245)	**0.036**	0.121 (-0.023, 0.265)	**0.021**	Low	0.105 (-0.025, 0.235)	0.060	0.109 (-0.033, 0.251)	**0.023**
	High	-0.076 (-0.193, 0.040)		-0.111 (-0.238, 0.015)		High	-0.063 (-0.181, 0.054)		-0.102 (-0.227, 0.024)	
rs2910164	Low	0.036 (-0.066, 0.139)	0.584	0.035 (-0.075, 0.146)	0.652	Low	0.049 (-0.052, 0.151)	0.753	0.068 (-0.041, 0.178)	0.808
	High	0.084 (0.000, 0.168)		0.079 (-0.011, 0.170)		High	0.085 (0.000, 0.170)		0.068 (-0.023, 0.159)	
rs2967615	Low	0.048 (-0.061, 0.157)	0.961	-0.001 (-0.123, 0.120)	0.548	Low	0.030 (-0.079, 0.139)	0.842	-0.030 (-0.147, 0.087)	0.308
	High	0.044 (-0.053, 0.142)		0.056 (-0.049, 0.161)		High	0.059 (-0.040, 0.157)		0.081 (-0.026, 0.189)	
rs303064	Low	0.027 (-0.081, 0.134)	0.941	0.013 (-0.107, 0.133)	0.708	Low	0.048 (-0.058, 0.154)	0.706	0.058 (-0.058, 0.174)	0.621
	High	0.028 (-0.066, 0.122)		0.033 (-0.069, 0.135)		High	0.013 (-0.082, 0.109)		-0.004 (-0.107, 0.100)	

rs34115976	Low	0.026 (-0.166, 0.219)	0.805	0.001 (-0.208, 0.210)	0.990	Low	0.040 (-0.148, 0.229)	0.552	0.038 (-0.170, 0.247)	0.550
	High	-0.037 (-0.218, 0.143)		-0.019 (-0.212, 0.175)		High	-0.070 (-0.257, 0.118)		-0.083 (-0.276, 0.110)	
rs35770269	Low	0.068 (-0.027, 0.164)	**0.026**	0.073 (-0.030, 0.177)	**0.021**	Low	0.064 (-0.031, 0.160)	**0.036**	0.063 (-0.043, 0.169)	**0.048**
	High	-0.085 (-0.176, 0.007)		-0.098 (-0.197, 0.000)		High	-0.077 (-0.169, 0.015)		-0.078 (-0.173, 0.017)	
rs3823658	Low	-0.003 (-0.138, 0.132)	0.473	0.023 (-0.123, 0.169)	0.564	Low	0.002 (-0.136, 0.140)	0.597	0.051 (-0.101, 0.202)	0.933
	High	0.053 (-0.065, 0.172)		0.071 (-0.056, 0.198)		High	0.045 (-0.071, 0.162)		0.044 (-0.076, 0.165)	
rs4294864	Low	-0.003 (-0.124, 0.117)	0.880	0.013 (-0.121, 0.147)	0.796	Low	-0.015 (-0.135, 0.105)	0.720	0.000 (-0.132, 0.133)	0.604
	High	0.010 (-0.103, 0.122)		0.04 (-0.081, 0.162)		High	0.022 (-0.092, 0.136)		0.061 (-0.061, 0.182)	
rs4919510	Low	0.013 (-0.078, 0.105)	0.375	-0.001 (-0.100, 0.098)	0.442	Low	0.024 (-0.069, 0.116)	0.596	0.002 (-0.100, 0.103)	0.445
	High	0.078 (-0.006, 0.162)		0.061 (-0.029, 0.152)		High	0.066 (-0.017, 0.149)		0.063 (-0.023, 0.150)	
rs55680837	Low	-0.015 (-0.112, 0.081)	0.424	-0.022 (-0.129, 0.085)	0.429	Low	-0.005 (-0.104, 0.093)	0.547	-0.010 (-0.118, 0.099)	0.660
	High	0.037 (-0.053, 0.127)		0.032 (-0.065, 0.129)		High	0.031 (-0.057, 0.119)		0.016 (-0.078, 0.109)	
rs56103835	Low	-0.020 (-0.120, 0.080)	0.550	-0.018 (-0.129, 0.093)	0.560	Low	-0.010 (-0.110, 0.090)	0.572	0.002 (-0.107, 0.111)	0.511
	High	-0.064 (-0.155, 0.027)		-0.068 (-0.167, 0.030)		High	-0.054 (-0.145, 0.037)		-0.064 (-0.163, 0.034)	
rs56863230	Low	-0.028 (-0.256, 0.200)	0.703	-0.030 (-0.284, 0.224)	0.630	Low	-0.022 (-0.247, 0.203)	0.931	0.019 (-0.220, 0.257)	0.681
	High	0.022 (-0.167, 0.210)		0.049 (-0.156, 0.254)		High	-0.002 (-0.195, 0.191)		-0.047 (-0.264, 0.171)	
rs60432575	Low	-0.137 (-0.307, 0.034)	0.519	-0.130 (-0.314, 0.055)	0.899	Low	-0.120 (-0.292, 0.052)	0.738	-0.171 (-0.365, 0.023)	0.338
	High	-0.046 (-0.210, 0.118)		-0.105 (-0.280, 0.071)		High	-0.070 (-0.233, 0.094)		-0.039 (-0.202, 0.124)	
rs60871950	Low	0.012 (-0.096, 0.120)	0.992	-0.007 (-0.124, 0.110)	0.877	Low	0.012 (-0.096, 0.119)	0.985	-0.024 (-0.144 ,0.097)	0.664
	High	0.009 (-0.093, 0.111)		-0.025 (-0.135, 0.084)		High	0.004 (-0.100, 0.108)		0.003 (-0.102, 0.108)	
rs61992671	Low	-0.031 (-0.235, 0.173)	0.933	-0.017 (-0.239, 0.204)	0.782	Low	-0.021 (-0.245, 0.202)	0.996	-0.080 (-0.346, 0.185)	0.471
	High	-0.012 (-0.185, 0.161)		0.031 (-0.154, 0.217)		High	-0.017 (-0.177, 0.142)		0.032 (-0.125, 0.190)	
rs6513497	Low	-0.024 (-0.177, 0.130)	0.395	-0.005 (-0.171, 0.161)	0.425	Low	-0.009 (-0.163, 0.145)	0.599	-0.002 (-0.179, 0.176)	0.589
	High	0.056 (-0.091, 0.203)		0.074 (-0.083, 0.232)		High	0.031 (-0.117, 0.178)		0.050 (-0.093, 0.193)	
rs67106263	Low	-0.115 (-0.254, 0.024)	0.193	-0.119 (-0.269, 0.032)	0.368	Low	-0.138 (-0.279, 0.004)	0.068	-0.155 (-0.310, 0.000)	0.083
	High	0.015 (-0.115, 0.145)		-0.026 (-0.165, 0.114)		High	0.033 (-0.094, 0.159)		0.013 (-0.120, 0.145)	
rs7210937	Low	0.043 (-0.051, 0.137)	0.602	0.04 (-0.062, 0.142)	0.624	Low	0.047 (-0.047, 0.142)	0.499	0.047 (-0.057, 0.151)	0.378

	High	0.014 (-0.071, 0.099)		0.012 (-0.079, 0.103)		High	0.015 (-0.070, 0.099)		0.007 (-0.081, 0.095)	
rs725980	Low	0.083 (-0.029, 0.196)	0.969	0.078 (-0.047, 0.203)	0.789	Low	0.078 (-0.035, 0.191)	0.987	0.101 (-0.023, 0.225)	0.762
	High	0.084 (-0.019, 0.188)		0.105 (-0.007, 0.217)		High	0.081 (-0.023, 0.185)		0.069 (-0.042, 0.181)	
rs73239138	Low	-0.040 (-0.144, 0.064)	0.497	-0.026 (-0.138, 0.087)	0.556	Low	-0.035 (-0.141, 0.070)	0.626	-0.027 (-0.143, 0.088)	0.546
	High	-0.016 (-0.103, 0.072)		-0.008 (-0.101, 0.086)		High	-0.023 (-0.107, 0.062)		0.003 (-0.086, 0.091)	
rs73410309	Low	0.145 (-0.044, 0.334)	0.523	0.150 (-0.055, 0.355)	0.571	Low	0.182 (-0.012, 0.376)	0.197	0.185 (-0.034, 0.405)	0.236
	High	0.030 (-0.137, 0.197)		0.028 (-0.151, 0.207)		High	0.012 (-0.148, 0.173)		0.006 (-0.156, 0.167)	
rs75330474	Low	-0.051 (-0.236, 0.135)	0.628	-0.076 (-0.277, 0.124)	0.464	Low	-0.077 (-0.307, 0.153)	0.598	-0.068 (-0.273, 0.137)	0.515
	High	0.041 (-0.180, 0.262)		0.065 (-0.171, 0.302)		High	-0.062 (-0.315, 0.192)		0.037 (-0.187, 0.262)	
rs77639117	Low	-0.060 (-0.293, 0.173)	0.690	-0.042 (-0.302, 0.218)	0.728	Low	0.173 (-0.419, 0.765)	0.858	-0.038 (-0.289, 0.213)	0.679
	High	-0.104 (-0.349, 0.142)		-0.083 (-0.349, 0.183)		High	-0.024 (-0.410, 0.362)		-0.031 (-0.317, 0.255)	
rs78861479	Low	0.162 (-0.432, 0.755)	0.562	0.200 (-0.458, 0.858)	0.405	Low	0.004 (-0.128, 0.136)	0.626	0.127 (-0.542, 0.797)	0.507
	High	-0.068 (-0.454, 0.318)		-0.126 (-0.545, 0.292)		High	-0.063 (-0.174, 0.048)		-0.089 (-0.492, 0.314)	
rs79402775	Low	0.003 (-0.127, 0.133)	0.487	0.018 (-0.123, 0.158)	0.739	Low	0.031 (-0.078, 0.141)	0.501	0.032 (-0.114, 0.178)	0.532
	High	-0.070 (-0.182, 0.043)		-0.024 (-0.146, 0.097)		High	0.059 (-0.039, 0.157)		-0.041 (-0.156, 0.075)	
rs7954025	Low	0.026 (-0.084, 0.135)	0.754	0.021 (-0.100, 0.143)	0.884	Low	0.079 (-0.038, 0.196)	0.756	0.007 (-0.114, 0.128)	0.485
	High	0.050 (-0.047, 0.147)		0.035 (-0.070, 0.140)		High	0.009 (-0.098, 0.117)		0.065 (-0.038, 0.169)	
rs8016027	Low	0.072 (-0.044, 0.189)	0.466	0.094 (-0.035, 0.224)	0.140	Low	0.023 (-0.081, 0.127)	0.395	0.100 (-0.025, 0.225)	0.135
	High	0.008 (-0.098, 0.115)		-0.04 (-0.156, 0.076)		High	0.026 (-0.064, 0.117)		-0.038 (-0.159, 0.082)	
rs878718	Low	0.035 (-0.066, 0.137)	0.817	0.075 (-0.037, 0.188)	0.464	Low	-0.052 (-0.237, 0.134)	0.900	0.031 (-0.082, 0.144)	0.737
	High	0.012 (-0.079, 0.103)		0.011 (-0.088, 0.110)		High	0.047 (-0.174, 0.269)		0.058 (-0.041, 0.157)	

[a] Z-Score standardization after ln-transformed.

[b] $P_{interaction}$ was calculated by modeling an interaction term of benzene CE levels (continuous) and SNP genotypes (categorical) into generalized linear models with adjustment for age, gender, smoking status, pack-years of smoking, drinking status, factory location, and BMI.

Significant levels of the associations, which was calculated by generalized linear models with adjustment for age, gender, smoking status, pack-years of smoking, drinking status, factory location, and BMI. were annotated as * $P_{trend} < 0.05$;

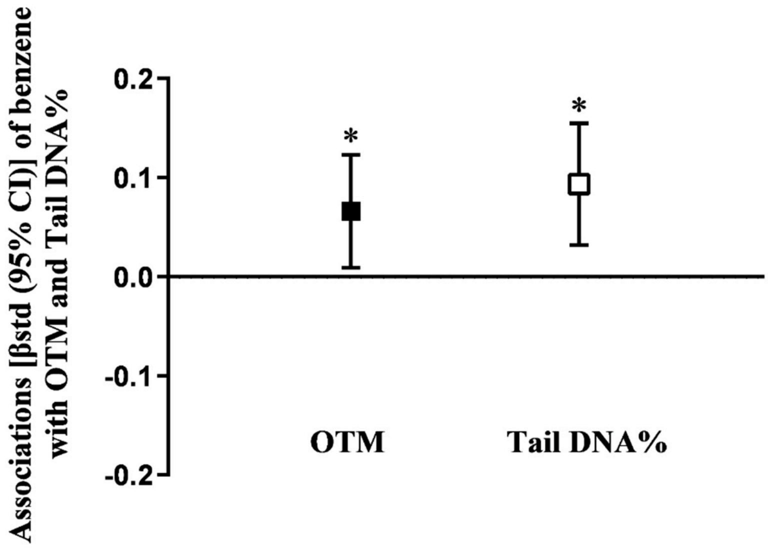

Fig. S1. Associations of BTX CE levels with OTM and Tail DNA% in subjects included in mirSNP analysis (n=667).

Note: Abbreviations: OTM, Olive tail moment; Tail DNA%, percent DNA in the Comet tail.

The lines represent β_{std} (95% CI) for the associations based on generalized linear models with adjustment for age, gender, smoking status, pack-years of smoking, drinking status, factory location, and BMI.

* P_{trend} <0.05.
